# A MicroRNA Network Dysregulated in Asthma Controls IL-6 Production in Bronchial Epithelial Cells

**DOI:** 10.1371/journal.pone.0111659

**Published:** 2014-10-31

**Authors:** Rocio T. Martinez-Nunez, Victor P. Bondanese, Fethi Louafi, Ana S. Francisco-Garcia, Hitasha Rupani, Nicole Bedke, Stephen Holgate, Peter H. Howarth, Donna E. Davies, Tilman Sanchez-Elsner

**Affiliations:** 1 Clinical and Experimental Sciences, Sir Henry Wellcome Laboratories, University of Southampton School of Medicine, Southampton General Hospital, Southampton, United Kingdom; 2 National Institute for Health Research (NIHR) Southampton Respiratory Biomedical Research Unit, Southampton Centre for Biomedical Research MP218, Southampton General Hospital, Southampton, United Kingdom; Virginia Tech University, United States of America

## Abstract

MicroRNAs are short non-coding single stranded RNAs that regulate gene expression. While much is known about the effects of individual microRNAs, there is now growing evidence that they can work in co-operative networks. MicroRNAs are known to be dysregulated in many diseases and affect pathways involved in the pathology. We investigated dysregulation of microRNA networks using asthma as the disease model. Asthma is a chronic inflammatory disease of the airways characterized by bronchial hyperresponsiveness and airway remodelling. The airway epithelium is a major contributor to asthma pathology and has been shown to produce an excess of inflammatory and pro-remodelling cytokines such as TGF-β, IL-6 and IL-8 as well as deficient amounts of anti-viral interferons. After performing microRNA arrays, we found that microRNAs -18a, -27a, -128 and -155 are down-regulated in asthmatic bronchial epithelial cells, compared to cells from healthy donors. Interestingly, these microRNAs are predicted *in silico* to target several components of the TGF-β, IL-6, IL-8 and interferons pathways. Manipulation of the levels of individual microRNAs in bronchial epithelial cells did not have an effect on any of these pathways. Importantly, knock-down of the network of microRNAs miR-18a, -27a, -128 and -155 led to a significant increase of IL-8 and IL-6 expression. Interestingly, despite strong *in silico* predictions, down-regulation of the pool of microRNAs did not have an effect on the TGF-β and Interferon pathways. In conclusion, using both bioinformatics and experimental tools we found a highly relevant potential role for microRNA dysregulation in the control of IL-6 and IL-8 expression in asthma. Our results suggest that microRNAs may have different roles depending on the presence of other microRNAs. Thus, interpretation of *in silico* analysis of microRNA function should be confirmed experimentally in the relevant cellular context taking into account interactions with other microRNAs when studying disease.

## Introduction

MicroRNAs (miRNAs) are small (20–26 nucleotides) non-coding RNAs that regulate gene expression post-transcriptionally, by binding to the 3′ UnTranslated Region (3′UTR) of their target mRNAs. MicroRNAs exert their effects via degradation or translation inhibition of their target mRNAs [1]. The effects of microRNAs are cell- and dose- dependent for both the miRNA and the target mRNA [2]–[4]; in addition, cooperation between different miRNAs has been shown to target gene networks [5] and influence disease [6], [7]. Thus, the study of miRNAs in cooperating networks is an emerging subject on the field of microRNAs [7]. However, excluding *in silico* analysis, few studies have explored the functional role of two or more microRNAs comparing individual versus groups of microRNAs [8].

MicroRNAs are subtle master controllers of gene expression and therefore excellent candidates for study in chronic multifactorial diseases such as asthma [9]. Asthma is an inflammatory disease of the respiratory airways and affects ∼300 million people worldwide [10]. The main features of asthma include bronchial airflow reversible obstruction with airway remodelling, impaired innate immunity to viral infections and dysregulated adaptive immune responses [11]. In asthma, inflammation and remodelling are intimately intertwined and the bronchial epithelium, initially thought of as a passive component, has progressively been gaining more attention [12]. Both at baseline and after stimulation, bronchial epithelial cells (BECs) from asthmatics have been shown to produce higher levels of cytokines such as TGF-β, IL-6 and IL-8 [13]–[16] that are also typically enriched in the lung of asthmatic patients [17], [18]. Therefore, the airway epithelium in asthma does not just suffer from the chronic inflammation, but actively influences it and contributes to the disease through the secretion of pro-inflammatory factors.

Using murine models, microRNAs such as miR-21, miR-126, miR-145 or miR-106a have been associated with allergic responses, potentially reflecting some features of human asthma [19]–[22]. The extensively studied miR-155 seems well placed to have an impact in asthma, encoded in a genomic region that maps to pollen sensitivity and asthma [23], [24]. Additionally, miR-155 deficient mice exhibit not only an impaired immune response but also dramatic airway remodelling similar to that present in asthmatic patients [25] Furthermore, we have shown in macrophages that miR-155 inhibits the TGF-β and IL-13 pathways [26], [27] which are important cytokines in airway remodelling and inflammation [18], [28].

However important miR-155 may be, it is but a single microRNA and we hypothesised that multiple microRNAs may be dysregulated in asthmatic epithelial cells and act as a master switch to some of the features that define this disease. We intended to test our hypothesis by using bioinformatics predictions together with experimental tools to validate the *in silico* data. We used asthmatic epithelial miRNA profiling as a model to explore potential co-operation of networks of microRNAs as opposed to single microRNAs in disease. Our data show that a network of microRNAs (miR-18a, miR-27a, miR-128 and miR-155) are down-regulated and may be involved in the activation of IL-6 and IL-8 gene transcription in the asthmatic bronchial epithelium. Importantly, our results strongly indicate that bioinformatics analysis should be validated by wet lab experiments. Additionally, our results suggest that microRNAs might have to be studied as co-operating networks, rather than in isolation, in order to accurately describe their biological role in a specific cellular context.

## Methods

### Ethics statement

Ethical approval was given by the Southampton and South Hampshire Research Ethics Committee (ethics numbers 05/Q1702/165 and 08/H0502/6). All subjects gave written informed consent following these guidelines.

### Human subjects

All subjects used in this study were non-smokers and were either healthy volunteer subjects or had asthma with a range of severity. Patient demographics are shown in Table S1. Asthma was defined as a physician's diagnosis of asthma with bronchial hyperresponsiveness or evidence of reversibility to salbutamol.

### Cell culture

Bronchial Epithelial Cells (BECs) were obtained by bronchial brushings upon fiber-optic bronchoscopy according to standard guidelines [29]. Bronchial brushings contained >95% epithelial cells and there was no significant difference in the proportion between healthy and asthmatic donors. BECs were cultured in collagen-coated T25 flasks in BEGM complete medium (Lonza) and were passaged when 80% confluent. Arrays and validations were performed using passage 1 cells.

BEAS2B cells [30] were maintained in RPMI 10% FBS and passaged when 80% confluent.

### MicroRNA microarrays

**Figure 1:**
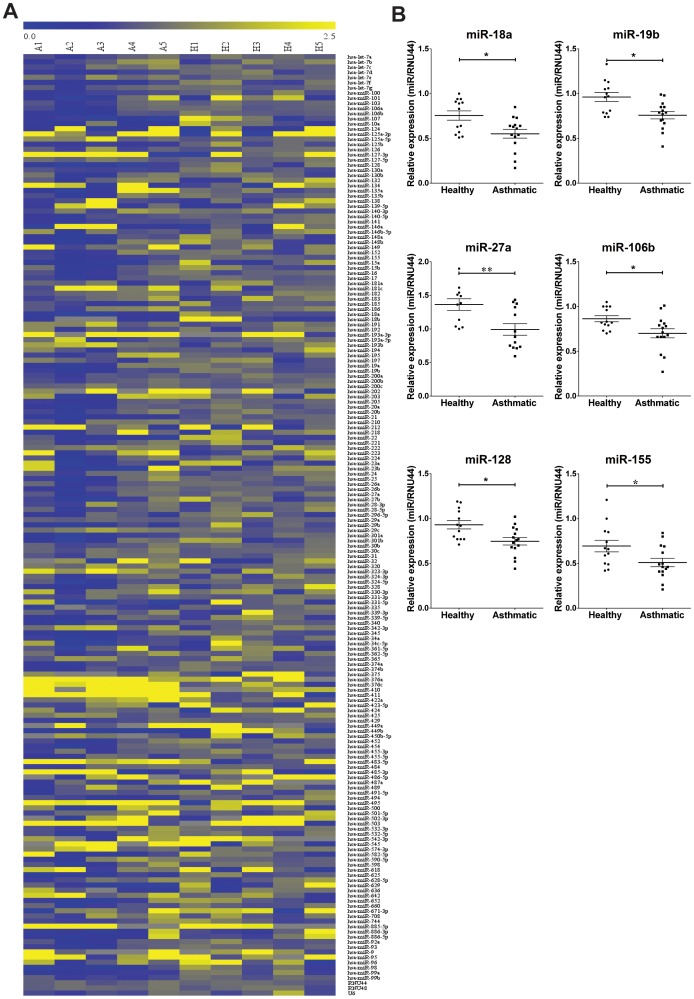
A network of microRNAs is dysregulated in asthmatic BECs. *A*: Heatmap showing fold expression of the microRNA array (n = 5 for healthy and asthmatic). Blue is down-regulated and yellow is up-regulated. *B*: Array validation. Expression of selected microRNAs was measured by TaqMan microRNA assays (n = 13 for healthy and n = 15 for asthmatics). ns = non-significant; * = p<0.05; ** = p<0.01, non-parametric unpaired t-test.

MicroRNA profiling of BECs was done using TaqMan Low Density Array Human Panel A (376 microRNAs in total and 3 controls, Applied Biosystems). We were able to detect the expression of 180 miRNAs (47.5%) in all the samples. MicroRNAs undetermined in more than two samples of the same type (healthy or asthmatic) were discarded. Controls used were RNU44, RNU48 and the small RNA U6. Relative expression was calculated using the Delta Delta Ct method [31]. We used RNU44 as reference gene since it showed the most consistent Ct (cycle threshold) values amongst all the donors (24.8376±0.1694 and 24.895±0.2113 for healthy and asthmatic, respectively). We used the average Delta Ct of the healthy controls as calibrator. Heatmap in Figure 1 was constructed using MeV [32]. The data discussed in this publication have been deposited in NCBI's Gene Expression Omnibus and are accessible through GEO Series accession number GSE61563.

### Transfections

BEAS2B were transfected with Interferin (Polyplus) following manufacturer's instructions. Antisense oligonucleotides against hsa-miR-18a, hsa-miR-27a, hsa-miR-128 and hsa-miR-155 were purchased from Ambion (Life Technologies). 25 nM of each anti-microRNA individually or in combination (100 nM total) were transfected using the appropriate amount of Anti-miR-Negative Control #1 as transfection control. Cells were harvested 24 h post-transfection for RT-qPCR and ELISA. In the case of TGF-β stimulation, cells were starved in 2% FBS following transfection. 24 h post-transfection cells were treated with 1 ng/mL TGF-β and harvested 24 h after stimulation for RT-qPCR analysis.

### Reverse transcription and real time PCR (RT-qPCR)

RNA extraction was performed using Tri Reagent (Ambion) following manufacturer's instructions. RNA was retro-transcribed into cDNA using High-Capacity cDNA Reverse Transcription Kit (Life Technologies) and qPCR was performed using TaqMan Gene Expression Assays (Life Technologies). For microRNA validations, TaqMan MicroRNA Assays were used following manufacturer's instructions.

### Luciferase experiments

pCDNA3.1_27a and pCDNA3.1_128. The genomic regions encompassing miR-27a and miR-128 were amplified (respectively) and separately cloned into pCDNA3.1. (-) (Invitrogen). MiR-27a was cloned between XbaI/BamHI sites and miR-128 between XhoI/HindIII sites. Primers employed were: miR-27a_HindIII-XbaI_FOR: AAGCTT TCTAGA AGA GAG GCC CCG AAG CCT GTG CC, miR-27a_BamHI_REV: GGATCC AGG GGA CAG GCG GCA AGG CC, miR-128_XhoI_FOR: CTCGAG CCT AGC TGT TTT CTG TGT AGC and miR-128_HindIII_REV: AAGCTT CTA TTT CTG AGT ATG ATG CAT G. pRLTK_WT_3′UTR_SMAD2 was generated previously by cloning the 3′UTR of human SMAD2 between XbaI and NotI sites of the pRLTK vector (Promega) [27]. pRLTK_MUT27a/128_3′UTR_SMAD2 vector containing a mutated version of the putative binding site for miR-27a and miR-128 between positions 8336–83430 was generated by site-directed mutagenesis on pRLTK_WT_3′UTR_SMAD2 using QuikChange Site-directed Mutagenesis Kit (Stratagene), following the manufacturer's instructions. Mutagenesis primers were: 3′UTR_MUT27a/128_SMAD2_FOR CCT TAG TGG CTG CAT CCT TGG TGC TCG AGG ATG GAG ATA TTA AAT GTG and 3′UTR_MUT27a/128_SMAD2_REV CAC ATT TAA TAT CTC CAT CCT CGA GCA CCA AGG ATG CAG CCA CTA AGG.

### Bioinformatics analysis

Pathway analysis for microRNAs was performed using DIANA microPath [33]. MicroRNA target predictions were performed using TargetScan and miRanda [34], [35]. miRecords [36] was also used to determine target-microRNA interaction in at least 4 of the 11 databases listed (miRanda, TargetScan, RNA hybrid, TargetScanCons). Further pathway analysis was performed based on bibliography and using the KEGG database.

### Statistical analysis

Statistical analysis was performed using GraphPad 6.0 software. For the array validation experiments a two-tailed Mann Whitney test was employed. In anti-microRNA transfections paired t-tests comparing to Anti-miRNA Negative controls for each experiment (n = 4 for individual transfections and n = 6 for simultaneous transfections) were done. ELISA experiments were analysed using a two-tailed non-parametric test (n = 4).

## Results

### A microRNA network is down-regulated in asthmatic bronchial epithelial cells

To test whether microRNAs were deregulated in the asthmatic bronchial epithelium, we performed Taqman Low Density Arrays using RNA from primary bronchial epithelial cells (BECs) isolated by bronchial brushing from healthy (n = 5) and asthmatic subjects (n = 5) (demographics shown in Table S1). Figure 1A is a representative heatmap of our results (complete dataset in Table S2) which showed 33 up-regulated and 91 down-regulated microRNAs (0.70≥fold [asthmatic/healthy]≥1.3), respectively. We validated our array data by performing specific RT-qPCR assays from BECs isolated from additional donors (13 healthy and 15 asthmatics). Figure 1B shows that we confirmed that microRNAs miR-18a, miR-19b, miR-27a, miR-106, miR-128 and miR-155 were down-regulated in asthmatic BECs compared to healthy counterparts while other microRNAs such as miR-34a, miR-93b, miR-140-5p, miR-152 and miR-374a) showed no differential expression (Figure S1.).

### Pathway analysis shows that dysregulation of miR-18a, miR-27a, miR-128 and miR-155 may affect TGF-β, IL-6, IL-8 and IFN pathways in the asthmatic bronchial epithelium

To identify cellular functions affected by the dysregulation in microRNA expression we performed *in silico* analysis. First, we used mirPath (DIANA-microT_v4.0, [37]). This program analyses the effect of individual microRNAs, the summative effect of a group of microRNAs or their intersection in genes targeted as well as pathways affected. We observed that miR-18a, miR-27a, miR-128 and miR-155 converged onto the TGF-β, MAPK, Jak-STAT, mTOR and cytokine-to-cytokine receptor signalling pathways involved in inflammation and remodelling. We decided to focus on these microRNAs and performed intersections of the candidate target genes predicted by DIANA. Venn diagrams of the overlapping genes for each pathway are shown in Figure S2. MiR-27a and miR-128 converged on the highest number of predicted target genes while the genes SMAD2, CDC42, RPS6KB1, RAP1B and SOS1 were predicted to be targeted by at least 3 of our candidate microRNAs (dataset in Table S3). These data suggested a function of this microRNA network (miR-18a, miR-27a, miR-128 and miR-155) in the inflammatory profile of asthmatic bronchial epithelial cells.

**Figure 2:**
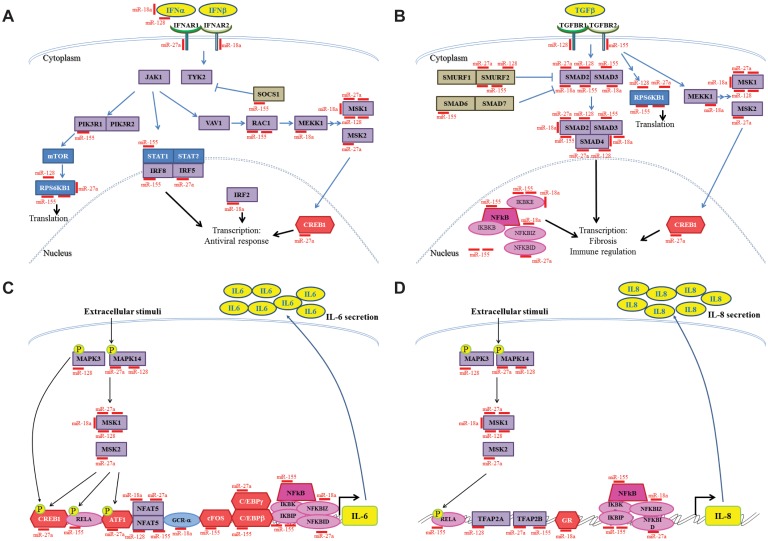
MiR-18a, miR-27a, miR-128 and miR-155 are predicted to target inflammatory pathways. *A*, *B*, *C*, and *D*: Interferon (IFN) alpha and beta signalling pathway, TGF-β signalling pathway, IL-6 and IL-8 secretion pathways, respectively, with the different microRNAs targeting effectors in each pathway.

We then narrowed our search taking into account the expression of targets in epithelium and their relevance to asthma and found out that the network of microRNAs showed predicted targets that mediate the TGF-β and IFN-α/IFN-β signalling pathways as well as mediators of IL-6 and IL-8 expression (Figure 2 A–D, respectively). Datasets for each pathway are present in Table S4. Together, our analysis suggested that down-regulation of miR-18a, miR-27a, miR-128 and miR-155 in the asthmatic bronchial epithelium may modulate inflammatory signalling and the TGF-β, IFN and IL-6/IL-8 pathways.

### MiR-18a, miR-27a, miR-128 and miR-155 have common predicted targets

**Figure 3:**
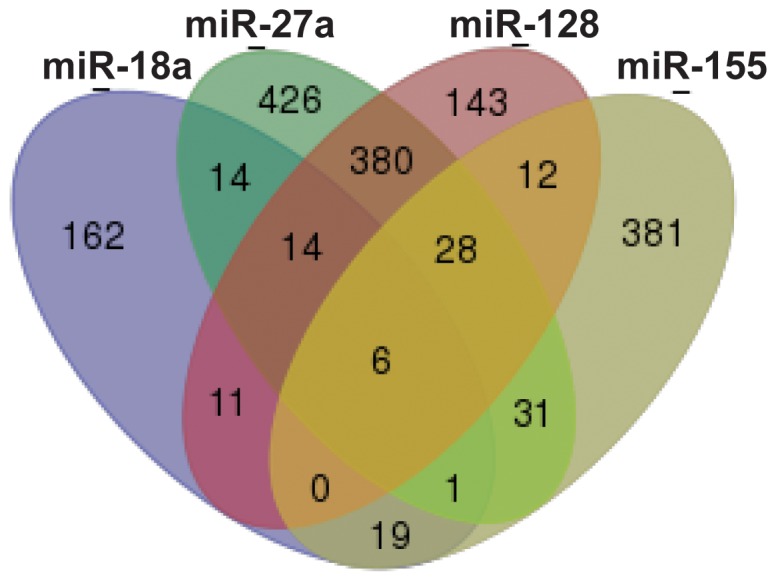
Mir-18a, miR-27a, miR-128 and miR-155 share predicted targets. Venn diagram showing the interaction between genes predicted to be targeted by the candidate microRNAs.

Following our pathway analysis, we used miRecords [36] to strengthen DIANA's *in silico* predictions. miRecords combines the datasets of several microRNA-target prediction tools. We took into account predicted targets for these microRNAs in miRanda, TargetScan, PITA and RNA Hybrid [35], [38]–[40] given that these tools consider different parameters when predicting microRNA seed pairing (conservation, stability of target-miRNA pairing and RNA secondary structure). Figure 3 is a Venn diagram showing that the intersection of the targets for these four microRNAs converges onto six genes, (MBNL2, KCNA1, QKI, SMAD2, PLAG1 and ATP2B1, Table S5), confirming the previous predictions of SMAD2 as a common target for the all the microRNAs in the network (Figure 2A and Table S4).

### Direct targeting of SMAD2 by miR-27a and miR-128

**Figure 4:**
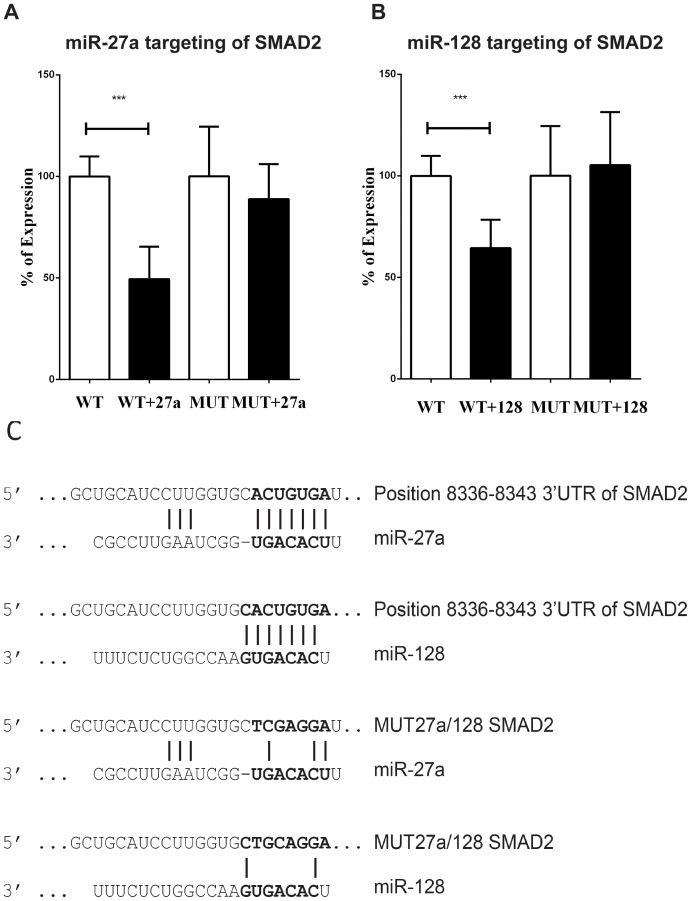
MiR-27a and miR-128 directly target the 3′UTR of SMAD2. *A* Renilla luciferase construct harbouring an SMAD2 3′-UTR fragment containing the predicted binding sites for miR-27a and miR-128 (wild type, WT) or its mutated version for both miRNAs binding (MUT) was co-transfected with either an empty expression vector, a miR-27a over-expressing vector (WT+27a and MUT+27a, respectively) or an miR-128 over-expressing vector (WT+128 and MUT+miR-128, respectively. One of three independent experiments is shown. Where no significance is shown = non-significant, ***p≤0.001. Error bars indicate Standard Error. *B* Schematic representing the binding region (and mutation site) of miR-27 and miR-128 in SMAD2 3′UTR.

MiR-18a and miR-155 have been previously shown to affect TGF-β activation by directly targeting SMAD2 [27], [41]. In order to test whether SMAD2 is also a direct target of miR-27a and miR-128 we performed renilla-luciferase reporter assays. The SMAD2 3′UTR predicted to be targeted by miR-27a and miR-128 was fused to a reporter renilla-luciferase gene and co-transfected with plasmids expressing miR-27 or miR-128. We used a Wild Type reporter vector (WT) and a mutated version (MUT) where the binding site (position 8336–8343) for both miR-27a and miR-128 was mutated by site directed mutagenesis (Figure 4C). Co-transfection of the WT vector with plasmids expressing either miR-27a or miR-128 showed a reduced luminescence, when compared to the samples co-transfected with control vectors (Figure 4A and, Figure 4B, respectively). As expected, the expression of the reporter containing the mutated version of SMAD2 3′UTR was not affected by co-transfection of miR-27a or miR-128 plasmids.

Taken together, these data confirmed the bioinformatics prediction that both miR-27a and miR-128 directly target SMAD2 3′UTR between positions 8336–8343.

### Effects of miR-18a, miR-27a, miR-128 and miR-155 individual modulation on TGF-β signalling in bronchial epithelial cells

Bioinformatics analysis and direct targeting assays suggested that mir-18a, miR-27a, miR-128 and miR-155 may regulate TGF-β effects in the bronchial epithelium through SMAD2 and other mediators (Figure 2, Figure 3, Figure 4 and [27], [41]). Due to the importance of the TGF-β pathway in remodelling in asthma [42], [43] we decided to determine the role of these microRNAs on the TGF-β pathway. We blocked their function by transfecting inhibitory oligonucleotides (anti-miRs) against each individual miRNA into human bronchial epithelial cells (BEAS2B) (Figure S3) and measured the effect on TGF-β signalling and mediators. Following the *in silico* predictions and experimental evidence regarding SMAD2 (Figure 2, Figure 3 and Figure 4), we expected that blocking individual microRNAs would enhance the TGF-β pathway by increasing the levels of SMAD2 but also of other targets such as SMAD3 and SMAD4 and the TGF-β Receptors (TGFBR 1 and TGFBR2) (see Figure 2A).

**Figure 5:**
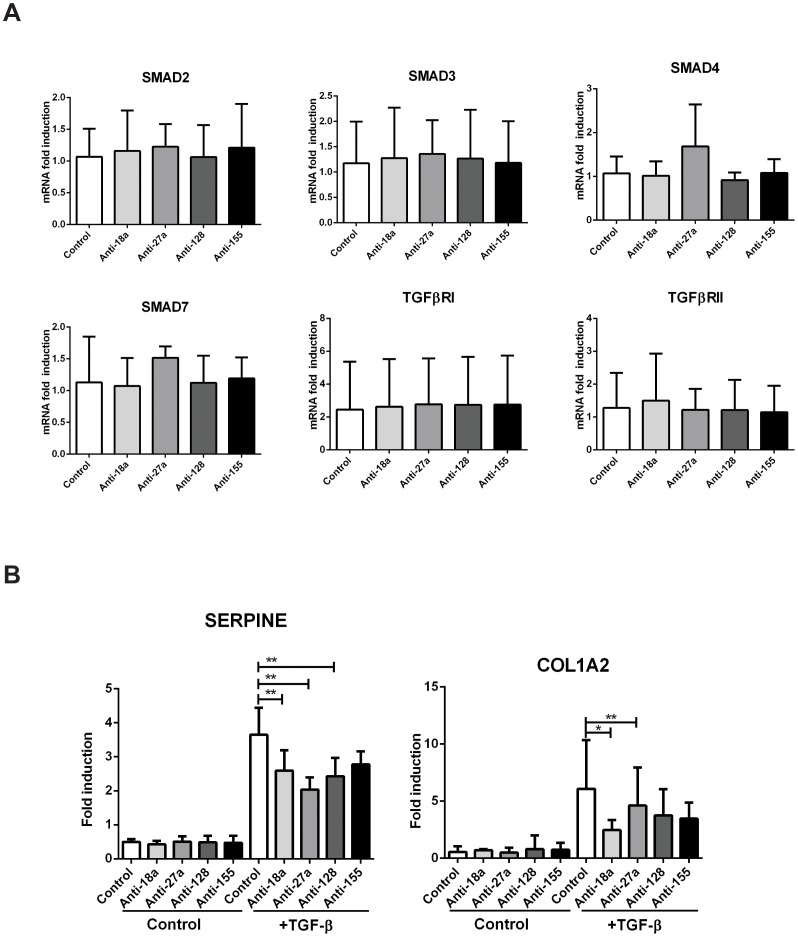
Individual microRNA modulatory effects on the TGF-β pathway. BEAS2B cells were transfected with anti-miRNA oligonucleotides against miR-18a, miR-27a, miR-128 and miR-155 or Anti-miRNA Control. *A*: Cells were harvested 24 h post-transfection. TGF-β effectors were analysed by RT-qPCR. *B*: 24 h post-transfection cells were stimulated with 1 ng/mL TGF-β and harvested 24 h later. Gene expression was analysed by RT-qPCR. No symbol: non-significant. n = 4 * = p<0.05; ** = p<0.01; *** = p<0.001, ratio paired t-test.

We first determined the mRNA expression of the TGF-β primary transduction pathway genes SMAD2, SMAD3, SMAD4 and SMAD7, as well as the TGFBR1 and TGFBR2 in BEAS2B cells transfected with anti-microRNA oligonucleotides individually targeting mir-18a, miR-27a, miR-128 and miR-155. MicroRNA inhibition seemed to have no effect on the mRNA expression of the TGF-β mediators assays (Figure 5A). We also determined protein levels of the direct target of all miRNAs SMAD2 by Western Blot. Although a modest trend was observed, no significant SMAD2 protein up-regulation when comparing specific anti-miRs to scrambled control was observed, with the exception of anti-miR-128 transfection (Figure S4).

In order to evaluate whether this minor effect (or any other effect on mediators that we had not considered) had any consequences on TGF-β signalling, we performed experiments where cells were transfected with anti-miR oligonucleotides and treated or not with 1 ng/mL TGF-β after 24 hours, harvesting after an additional 24 hours for RT-qPCR analysis. We quantified the mRNA expression of two TGF-β dependent genes: COL1A2 (collagen type 1 alpha 2) and SERPINE1 (Plasminogen Activator Inhibitor Type 1) [44], [45]. As expected, TGF-β treatment led to up regulation of SERPINE1 and COL1A2 mRNA expression. However inhibition of miRNAs individually led to an unexpected mild decrease in SERPINE1 mRNA levels upon TFG-β stimulation compared to stimulated control anti-miR transfected cells (Figure 5B left panel). COL1A2 expression was significantly down regulated by anti-miR-18a and -27a (Figure 5B right panel).

Taken together, our results suggested that despite strong bioinformatics predictions and preliminary luciferase assays in HeLa cells, individual microRNAs down-regulated in asthmatic epithelium do not increase SMAD2 levels or TGF-β activity in human bronchial epithelial cells.

### Effects of individual modulation of miR-18a, miR-27a, miR-128 and miR-155 on the inflammatory profile of bronchial epithelial cells

**Figure 6:**
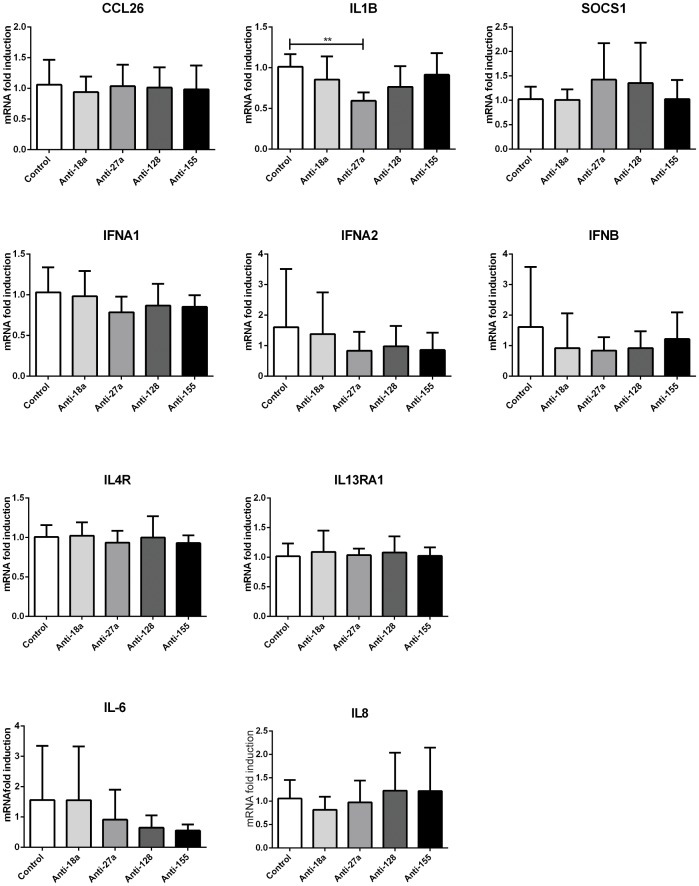
Individual microRNA modulatory effects on inflammatory genes. BEAS2B cells were transfected with anti-miRNA oligonucleotides against miR-18a, miR-27a, miR-128 and miR-155 or Anti-miRNA Control. Cells were harvested 24 h post-transfection. Inflammatory genes were analysed by RT-qPCR. No symbol: non-significant. n = 4 * = p<0.05; ** = p<0.01, ratio paired t-test.

Our *in silico* data also suggested a role for miR-18a, miR-27a, miR-128 and miR-155 (Figure 2 and Figure S2) in the inflammatory profile of the airway epithelium of asthmatics. Asthmatic epithelium shows an exacerbated inflammatory status with over-expression of cytokines such as IL-6 and IL-8 that play also an important role in remodelling [17], [46], [47]. Interestingly, several cytoplasmic and nuclear factors involved in the regulation of IL-6 and IL-8 were predicted targets of the network of microRNAs (Figure 2C and Figure 2D). Also, several inflammatory mediators, of potential relevance to asthma, such as SOCS1, IL1B, IL13RA1 and CCL26 [48]–[51] could be potentially affected, directly or indirectly by one or more microRNAs of this list. One interesting possibility was that microRNA down-regulation would result in an increase of these cytokines and result in augmented inflammation. We thus analysed the effects of individual inhibition of miR-18a, miR-27a, miR-128 and miR-155 on the expression of inflammatory mediators by RT-qPCR expecting to see an up-regulation of inflammatory cytokines and chemokines such as IL1B, CCL26, IL-6 and IL-8 or down regulation in antiviral IFNs. Figure 6 shows that only inhibition of miR-27a led to a modest decrease of IL1β mRNA expression. MicroRNA modulation showed no effect on any of the other candidate genes tested.

We therefore concluded that modulation of the individual microRNAs miR-18a, miR-27a, miR-128 and miR-155 does not seem to significantly alter the expression of inflammatory mediators in human bronchial epithelial cells.

### Effects of simultaneous down-regulation of microRNAs on the TGF-β pathway in bronchial epithelial cells

Our results so far showed that modulation of individual candidate microRNA (Figure 1B) levels did not have an effect on the TGF-β pathway or on the expression of inflammatory mediators in bronchial epithelial cells (Figure 5 and Figure 6) in opposition to our preliminary analysis (Figure 2, Figure 3 and Figure 4). We wondered whether transfection of an individual microRNA may have a knock-on effect on the rest of microRNAs studied explaining this contradiction. We assessed the effects of inhibition of miR-18a, miR-27a, miR-128 and miR-155 on the expression of the other miRNAs by qPCR, showing that individual transfection increased the levels of the rest of microRNAs (Figure S5). These results suggest that, in order to exert an effect on the pathways studied, we might need to knock-down all the microRNAs studied simultaneously. Taking these results into account and published data showing that microRNAs are able to work cooperatively [7], we hypothesised that the effects of microRNA dysregulation may be different when considered simultaneously, as opposed to individually.

To test this hypothesis we transfected BEAS2B with a pool of anti-miRs against miR-18a, miR-27a, miR-128 and miR-155 (hence forth Anti-MiX), in order to diminish their expression simultaneously (Figure S6). We expected that this simultaneous knock-down could confirm the *in silico* prediction of an enhancement of the TGF-β pathway (Figure 2A).

**Figure 7:**
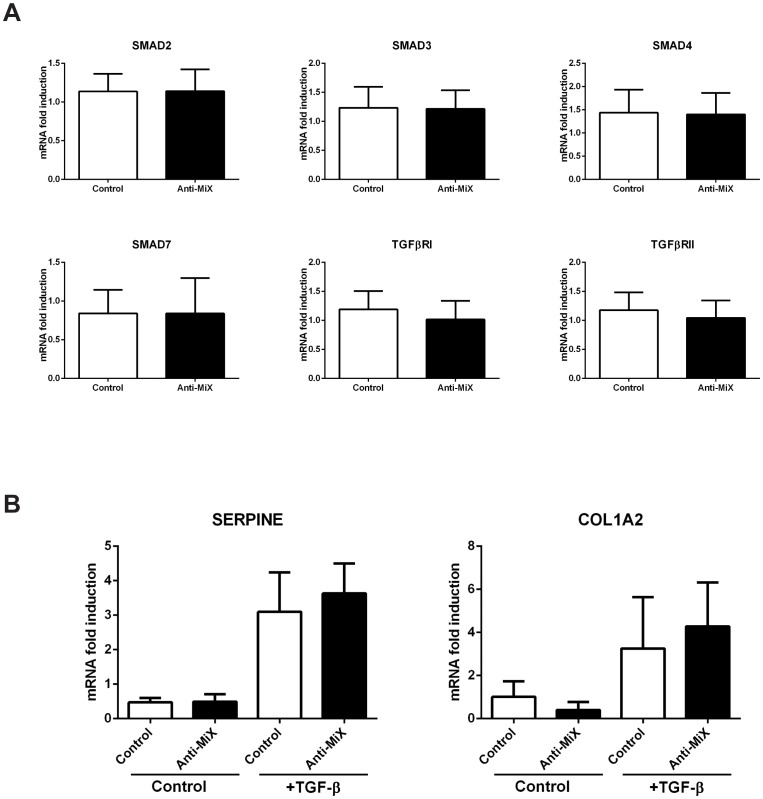
MicroRNA network modulatory effects on the TGF-β pathway. BEAS2B cells were transfected with anti-miRNA oligonucleotides against miR-18a, miR-27a, miR-128 and miR-155 together (Anti-MiX) or Anti-miRNA Control. *A*: Cells were harvested 24 h post-transfection. TGF-β effectors were analysed by RT-qPCR *B*: 24 h post-transfection cells were stimulated with 1 ng/mL TGF-β and harvested 24 h later. Gene expression was analysed by RT-qPCR. n = 6. No symbol = non-significant * = p<0.05, ratio paired t-test.

We first analysed the effect of microRNA down-regulation on the TGF-β signalling cascade components. Figure 7A shows that the simultaneous down-regulation of the pool of microRNAs in BEAS2B cells had no effect on the mRNA levels of the TGF-β signal transducers analysed. We also evaluated the levels of SMAD2 protein by western blot in BEAS2B cells transfected with Anti-MiX as compared to control and observed no differences (Figure S7).

To investigate whether TGF-β signalling could be affected through other untested mediators, we determined the levels of TGF-β-dependent genes after TGF-β stimulation in BEAS2B cells transfected with Anti-MiX. Figure 7B shows that Anti-MiX transfection had no effect on the TGF-β dependent activation of SERPINE1 and COL1A2 mRNA expression. Thus, our data suggested that the pool of microRNAs does not modulate the activity of TGF-β or the levels of TGF-β pathway transducers in the bronchial epithelium.

### Effects of miR-18a, miR-27a, miR-128 and miR-155 simultaneous down-regulation on the inflammatory profile of bronchial epithelial cells

**Figure 8:**
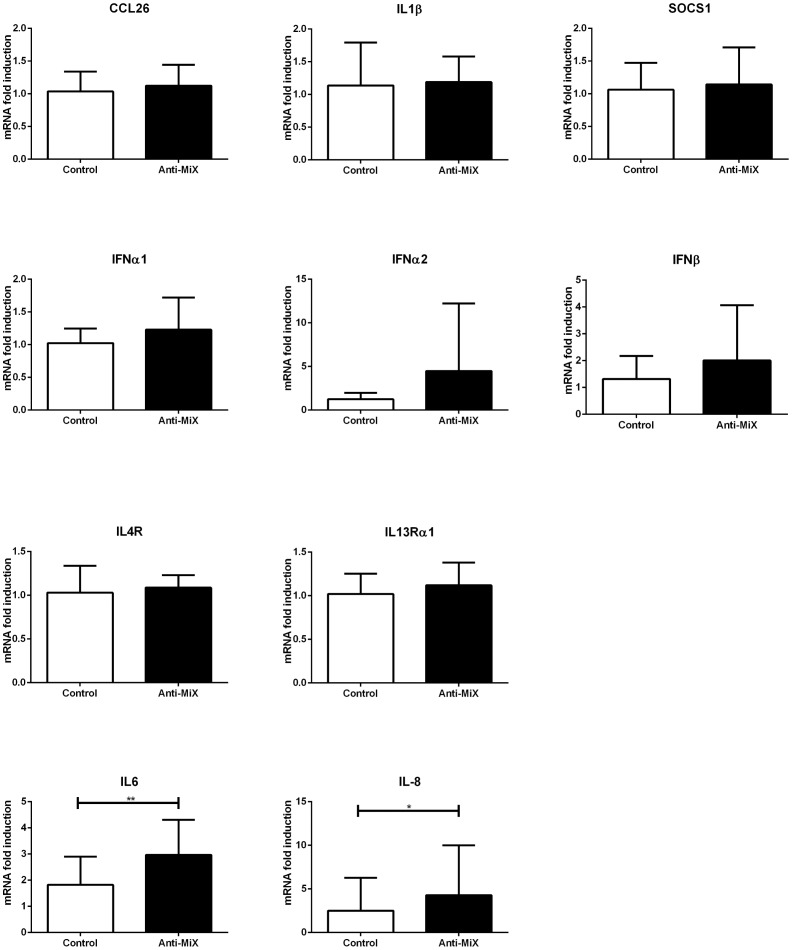
A microRNA network modulates IL-6 and IL-8 mRNA expression. BEAS2B cells were transfected with anti-miRNA oligonucleotides against miR-18a, miR-27a, miR-128 and miR-155 together (Anti-MiX) or Anti-miRNA Control. Cells were harvested 24 h post-transfection. Inflammatory genes were analysed by RT-qPCR. n = 6. No symbol: non-significant. * = p<0.05; ** = p<0.01, ratio paired t-test.

After our initial set of results, we wondered whether this network of microRNAs had a different effect on inflammatory mediators when co-operating compared to that exerted by the microRNAs individually (Figure 6). We therefore analysed the effects of the simultaneous down-regulation of our candidate microRNAs on the inflammatory mediators included in Figure 6. We found that simultaneous down-regulation of miR-18a, miR-27a, miR-128 and miR-155 in bronchial epithelial cells significantly increased both interleukin IL-6 and IL-8 mRNA levels (Figure 8). None of the other genes tested (CCL26, IL1B, SOCS1, IFNA1, IFNA2, IFNB, IL13RA1, IL4) showed significant differences (Figure 8).

Our results suggest that miR-18a, miR-27a, miR-128 and miR-155 work as a co-operative network, and that simultaneous down-regulation of this network in asthmatic epithelium may lead to an over expression of IL-6 and IL-8.

### MicroRNA inhibition modulates the secretion of IL-6 in human bronchial epithelial cells

MicroRNAs act post-transcriptionally by degrading mRNA and also by blocking mRNA translation. Our results so far showed that the network of microRNAs – and not the individual microRNAs separately- was regulating the expression of IL-6 and IL-8 at the mRNA level (Figure 8). To confirm these findings at the protein level we assayed secretion of IL-6 and IL-8 by ELISA on the supernatant of BEAS2B cells transfected with inhibitors of the candidate miRNAs individually or as a network.

**Figure 9:**
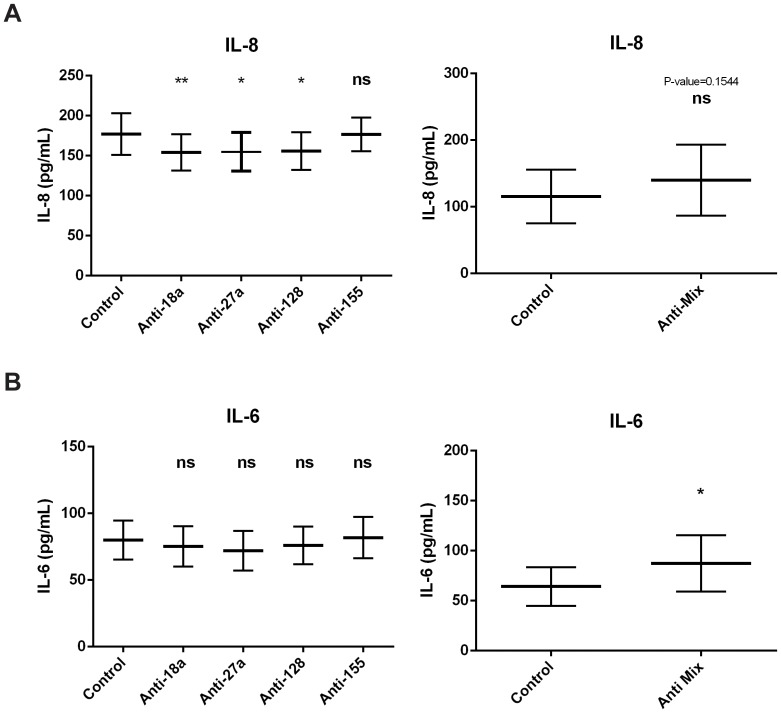
A microRNA network affects IL-6 secretion in bronchial epithelial cells. BEAS2B cells were transfected with anti-miRNA oligonucleotides against miR-18a, miR-27a, miR-128 and miR-155 separately or together (Anti-MiX) or Anti-miRNA Control. Cells were harvested 24 h post-transfection. IL-8 (*A*) and IL-6 (*B*) secretion was measured by ELISA. n = 4 ns: non-significant. * = p<0.05; ** = p<0.01, ratio paired t-test.

Figure 9A (left) shows that inhibition of individual microRNAs led to a significant decrease of IL-8 secretion in all cases except for miR-155 inhibition, while the pool of microRNAs seemed to produce a non-significant increase on IL-8 secretion (p = 0.1544, Figure 9A right panel). Inhibition of individual microRNAs did not alter the secretion of IL-6 (Figure 9B left panel). Importantly, when all microRNAs where inhibited together, we observed a significant increase in the secretion of IL-6 cytokine (Figure 9B, right panel).

Taken together our results show that microRNAs, acting as a network, may show opposite effects to individual microRNAs. In our study, the functional role of the network may explain the increased secretion of important inflammatory cytokines in asthma, such as IL-6 or IL-8, while the individual microRNAs do not seem to be involved in any of the potentially pathological pathways tested (remodelling or inflammation).

## Discussion

In this study we show a novel and relevant role for a network of microRNAs as the possible molecular mechanism underlying IL-6- and IL-8-mediated inflammation in the asthmatic epithelium. We make an important observation of broader consequences in the study of microRNAs in disease: microRNAs may have different and even opposite effects when co-operating in groups rather than when acting individually.

We found that miR-18a, mir-27a, miR-128 and miR-155 were down-regulated in BECs from asthmatics compared to healthy donors (Figure 1). Using several bioinformatics tools, we predicted that these microRNAs may play a role in airway inflammation and remodelling by targeting multiple mRNAs and affecting inflammatory mediators important in asthma such as TGF-β, IFNs, IL-6 and IL-8 (Figure 2 and Figure S2) [15], [17], [18], [52].

Regardless of our assays showing direct targeting (Figure 4), published evidence in other cell types [27], [41] and strong *in silico* predictions (Figure 2 and Figure 3) neither the individual nor the simultaneous inhibition of these four microRNAs in human bronchial epithelial cells increased the downstream signalling of TGF-β (Figure 5 and Figure 7). Additionally, potential inflammatory targets seemed to be equally unaffected when microRNAs were knocked-down individually (Figure 6). These data underline the importance of wet lab experiments in the relevant cellular model to validate bioinformatics predictions. Interestingly, simultaneous down-regulation of the four miRNAs led to an increase in IL-6 and IL-8 mRNA and IL-6 protein expression in human bronchial epithelial cells (Figure 8 and Figure 9). Over-expression of IL-8 has been long associated with asthma and inflammation [53] and it is thought to cause neutrophil recruitment, which is one of the hallmarks of virus-induced asthma exacerbations [54]. IL-6 has been inversely correlated with lung function in asthmatics [17] and in mice exposure to allergen can trigger the production of this cytokine [55]. Our data suggest that deficiency of microRNAs may contribute to the exacerbated expression of both IL-6 and IL-8 in asthma.

Our analysis suggests that the microRNA network may exert its effect not through a single target but a combination of targets such as transcription factors NFATs, NFKB, CREB1, CEBPB, CEBPA, GR, RELA (Figure 2 and Table S4) and signalling transducers MAPK and mTOR that may contribute to the regulation of IL-6 and IL-8 expression (Table S3). Thus, simultaneous down-regulation of the microRNA network but not the individual microRNAs may cause mild disruptions to different signalling pathways that, by accumulation, might lead to the over-expression of IL-6 and IL-8 mRNA (Figure 8).

There are several studies that are in agreement with our data. For example, Forrest et al identified four microRNAs up-regulated during monocyte differentiation and showed that their simultaneous over-expression triggers specific pro-differentiative changes not elicited by the over-expression of the individual microRNAs. Interestingly, two of those miRNAs would also target a microRNA with anti-differentiative role [56]. Chang et al showed that none of the individual microRNAs studied were able to suppress CD3-triggered proliferation of CD4+ T cells, while this was achieved by microRNAs working in groups [8]. Also, Yao et al showed how miR-155 affects SOCS1 but not some of the other confirmed targets SMAD2 and SMAD5 [57]. This co-operative function may be due to reprogramming of mRNA targeting by microRNAs by affecting the secondary structure of the 3′UTR of their targets [39], [58]. It is also possible that changes in miRNA levels may affect the expression of one or more microRNAs, or modify the levels – and thus affect avidity- of their target mRNAs [59], [60]. Also, competition for Argonaute proteins that are essential to the RISC complex and to microRNA activity [61] may contribute to this reprogramming of microRNA function [62].

It is important to note that we do not rule out the role of other microRNAs that may change the function of the microRNA network. Our data suggests that individual modulation of specific miRNAs may affect the levels of the other miRNAs (Figure S5), with consequences that are difficult to predict. Additionally, the exact levels of microRNAs in the cell may be essential to their role; we have manipulated microRNA expression but the down-regulation achieved may not be an exact mimic of the actual microRNA levels in the diseased cells. Our own data preclude us from pursuing a reliable molecular mechanism of action for the increase in IL-6 and IL-8 mRNA expression; miR-128 targets SMAD2 3′UTR directly (Figure 4) and anti-miR-128 increases SMAD2 protein (Figure S4), however, miR-128 down-regulation seems to reduce TGF-β signalling rather than increase it (Figure 5B). Regardless of these limitations, this network of microRNAs may potentially be used as a therapy to balance the levels of IL-6 and IL-8 in asthma with a low off-target effect (as shown in Figure 5, Figure 6, Figure 7, Figure 8 and Figure 9). In vivo work and then pre-clinical testing would be needed to validate a potential benefit to asthmatic patients.

In summary, we present an important and novel finding: microRNAs miR-18a, miR-27a, miR-128 and miR-155 work as a network and regulate the expression of IL-6 and IL-8 in the bronchial epithelium. Additionally, our work demonstrates the need of experimental testing of *in silico* predictions and the importance of considering the role of several microRNAs simultaneously when assessing the effects of miRNAs in disease.

## Supporting Information

Figure S1 **MicroRNAs not validated in the BECs array.**
(JPG)

Figure S2 **Venn diagrams showing the intersection of the number of candidates in pathways predicted to be targeted by miR-18a, miR-27a, miR-128 and miR-155.**
(JPG)

Figure S3 **Transfection efficiency of individual anti-miRs against miR-18a, miR-27a, miR-128 and miR-155.**
(JPG)

Figure S4 **Effects of individual down-regulation of miR-18a, miR-27a, miR-128 and miR-155 in SMAD2 protein levels.**
(JPG)

Figure S5 **Effects of individual down-regulation of miR-18a, miR-27a, miR-128 and miR-155 on the expression levels of the candidate microRNAs.**
(TIF)

Figure S6 **Transfection efficiency of pooled anti-miRs against miR-18a, miR-27a, miR-128 and miR-155.**
(JPG)

Figure S7 **Effects of simultaneous down-regulation of miR-18a, miR-27a, miR-128 and miR-155 in SMAD2 protein levels.**
(JPG)

Table S1 **Demographics of the population employed for microRNA profiling of human bronchial epithelial cells.**
(DOCX)

Table S2 **Bronchial epithelial cells microRNA array results.**
(DOCX)

Table S3 **Candidate genes predicted to be targeted in each signalling pathway.**
(DOCX)

Table S4 **Candidate genes predicted to be targeted in TGF-β and IFNs signalling pathway as well as IL-6 and IL-8 secretion pathways.**
(DOCX)

Table S5 **Complete set of candidate genes predicted to be targeted by miR-18a, miR-27a, miR-128 and miR-155.**
(DOCX)
