# Supplementary figures and images for: A MicroRNA Network Dysregulated in Asthma Controls IL-6 Production in Bronchial Epithelial Cells
Source: PLoS One. 2014 Oct 31;9(10):e111659. doi: 10.1371/journal.pone.0111659 (PMC4216117; doi:10.1371/journal.pone.0111659)

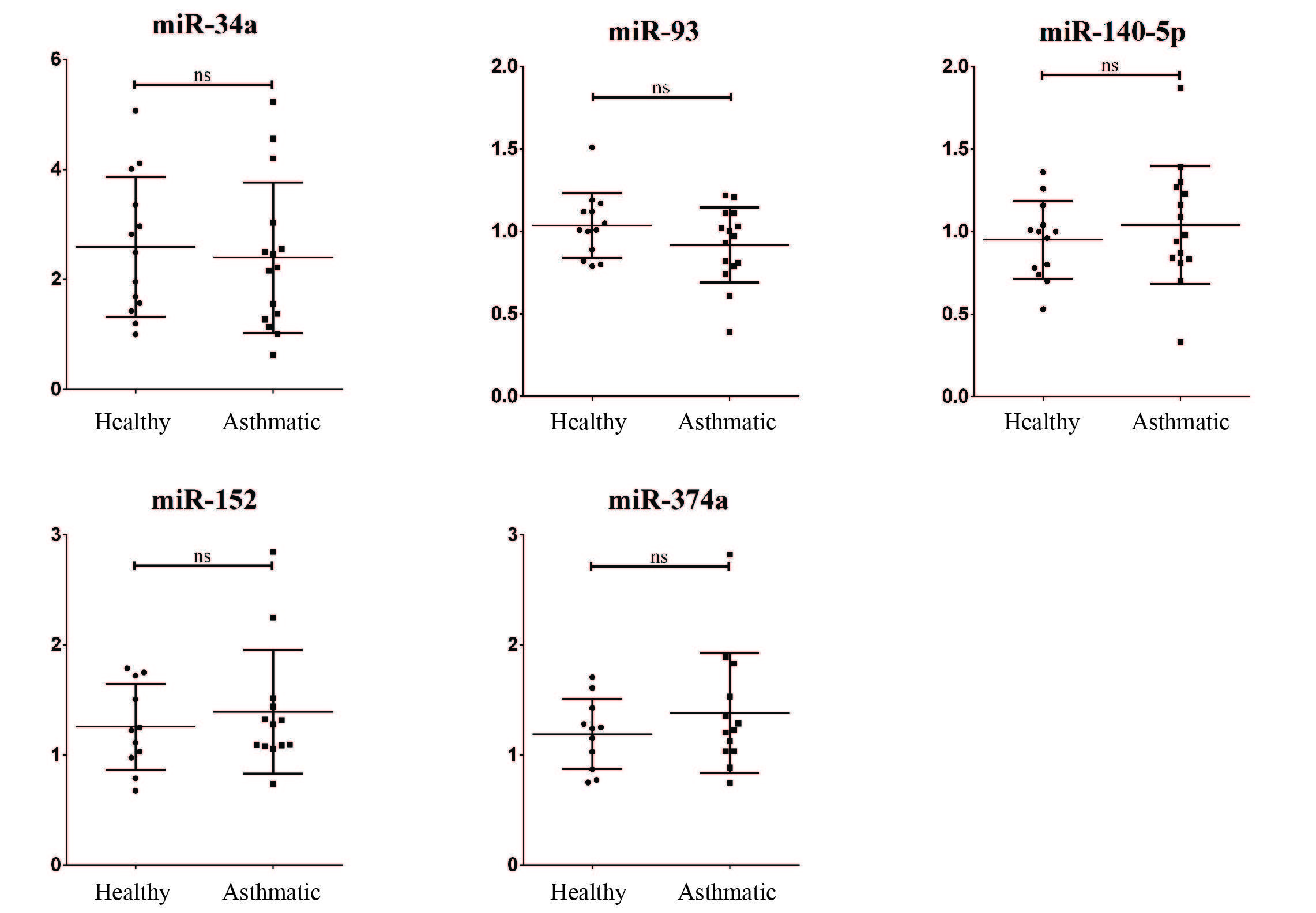

Supplement: Figure S1 — MicroRNAs not validated in the BECs array. (JPG) [file pone.0111659.s001.jpg]

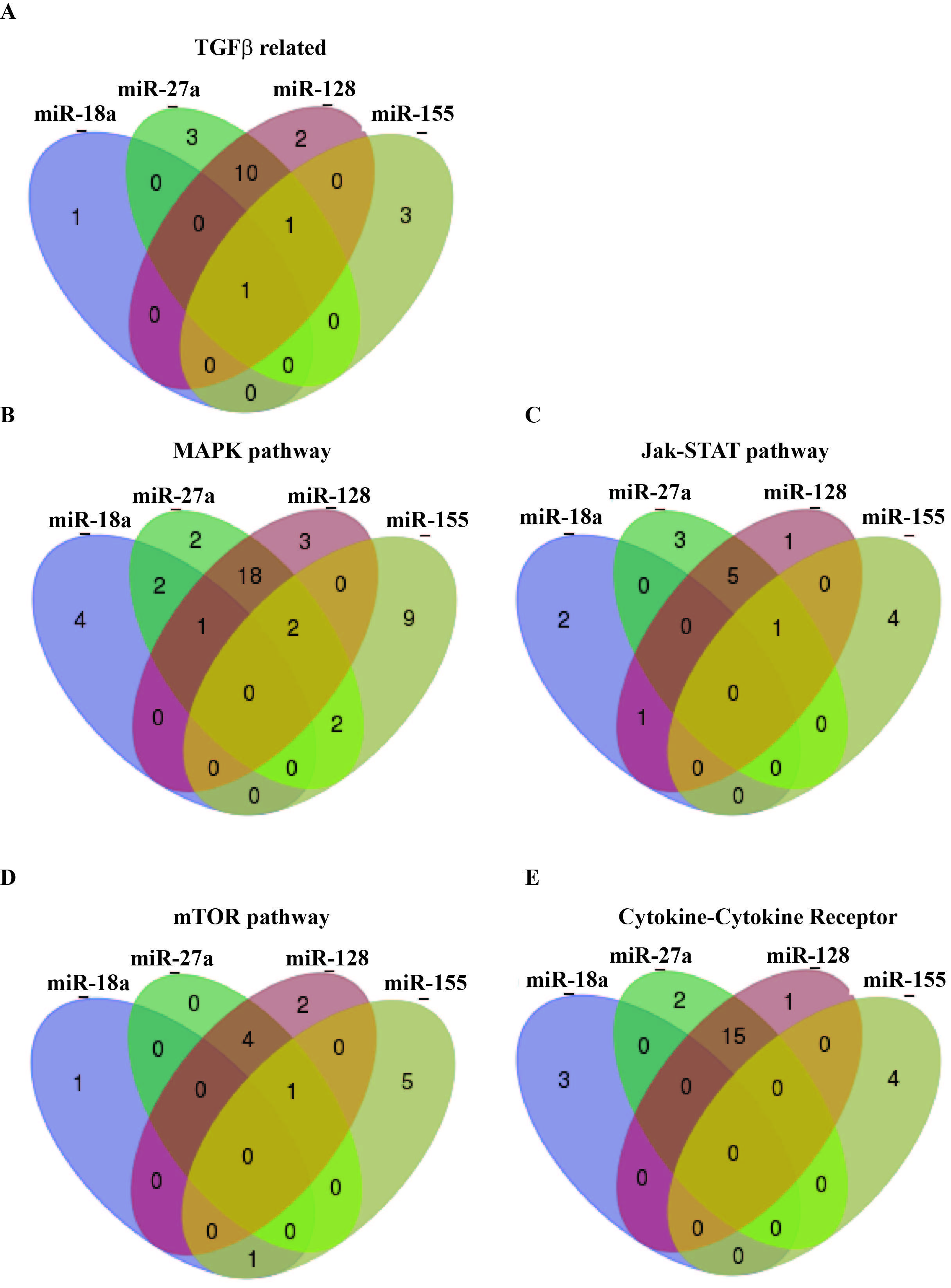

Supplement: Figure S2 — Venn diagrams showing the intersection of the number of candidates in pathways predicted to be targeted by miR-18a, miR-27a, miR-128 and miR-155. (JPG) [file pone.0111659.s002.jpg]

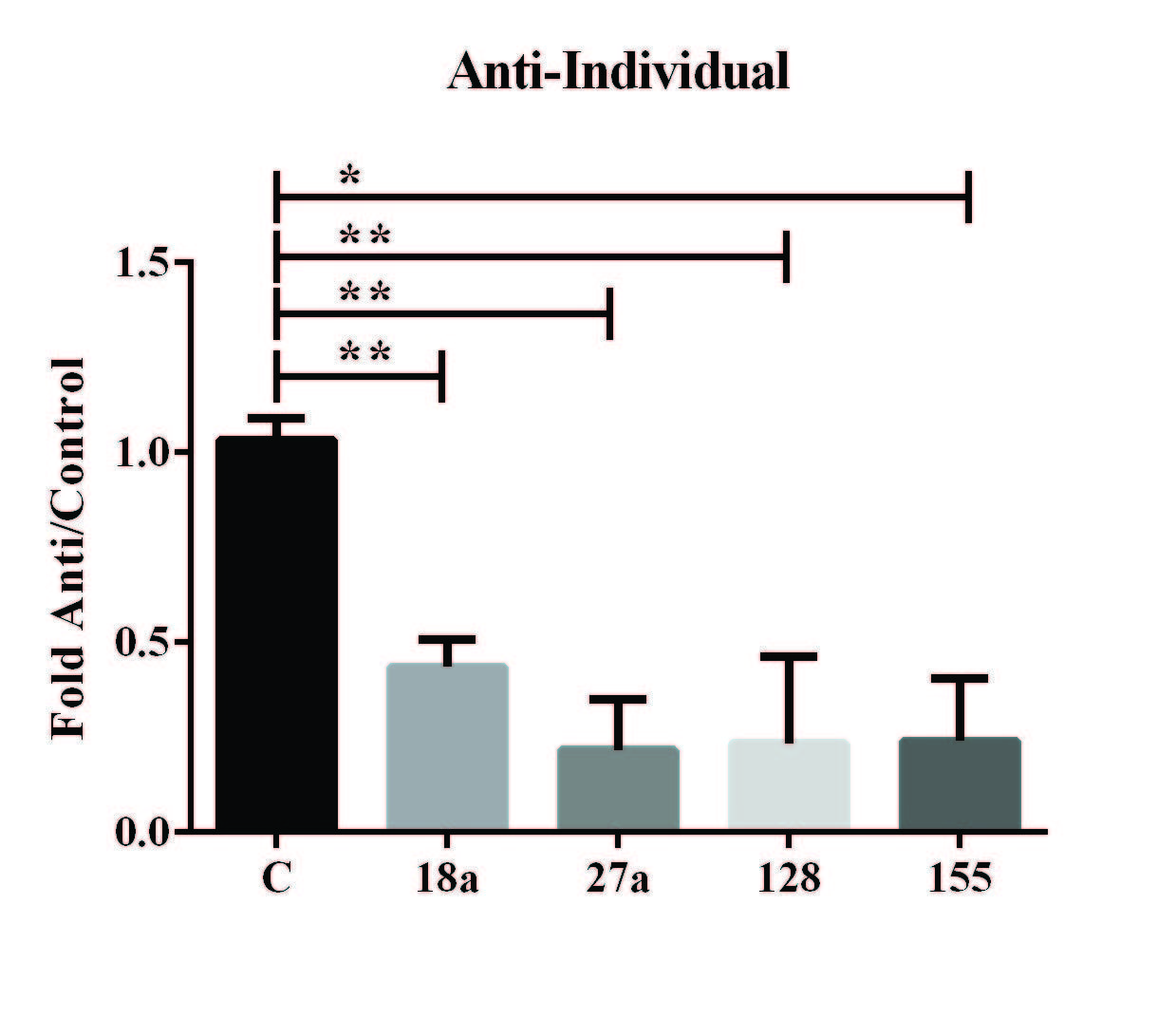

Supplement: Figure S3 — Transfection efficiency of individual anti-miRs against miR-18a, miR-27a, miR-128 and miR-155. (JPG) [file pone.0111659.s003.jpg]

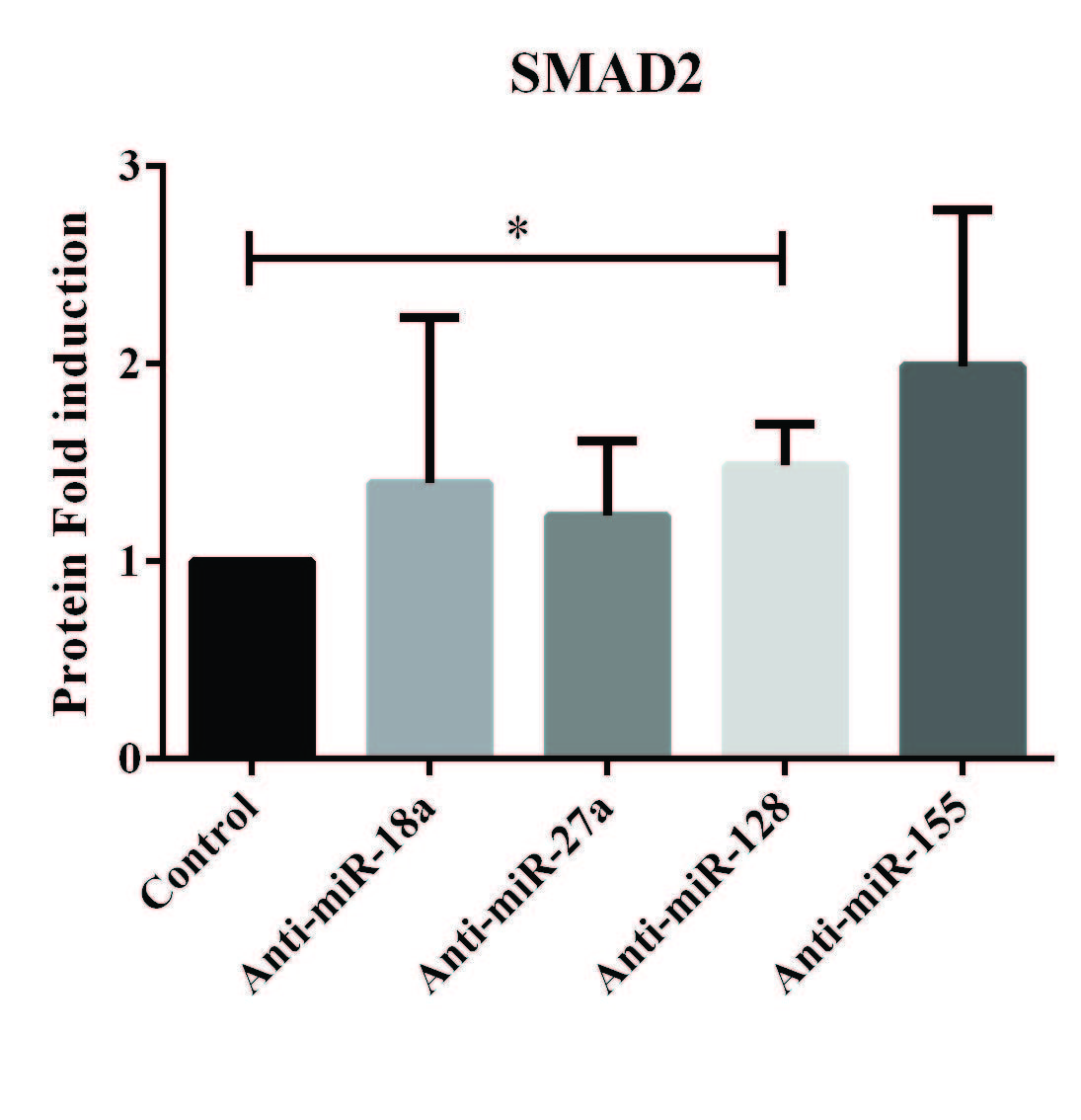

Supplement: Figure S4 — Effects of individual down-regulation of miR-18a, miR-27a, miR-128 and miR-155 in SMAD2 protein levels. (JPG) [file pone.0111659.s004.jpg]

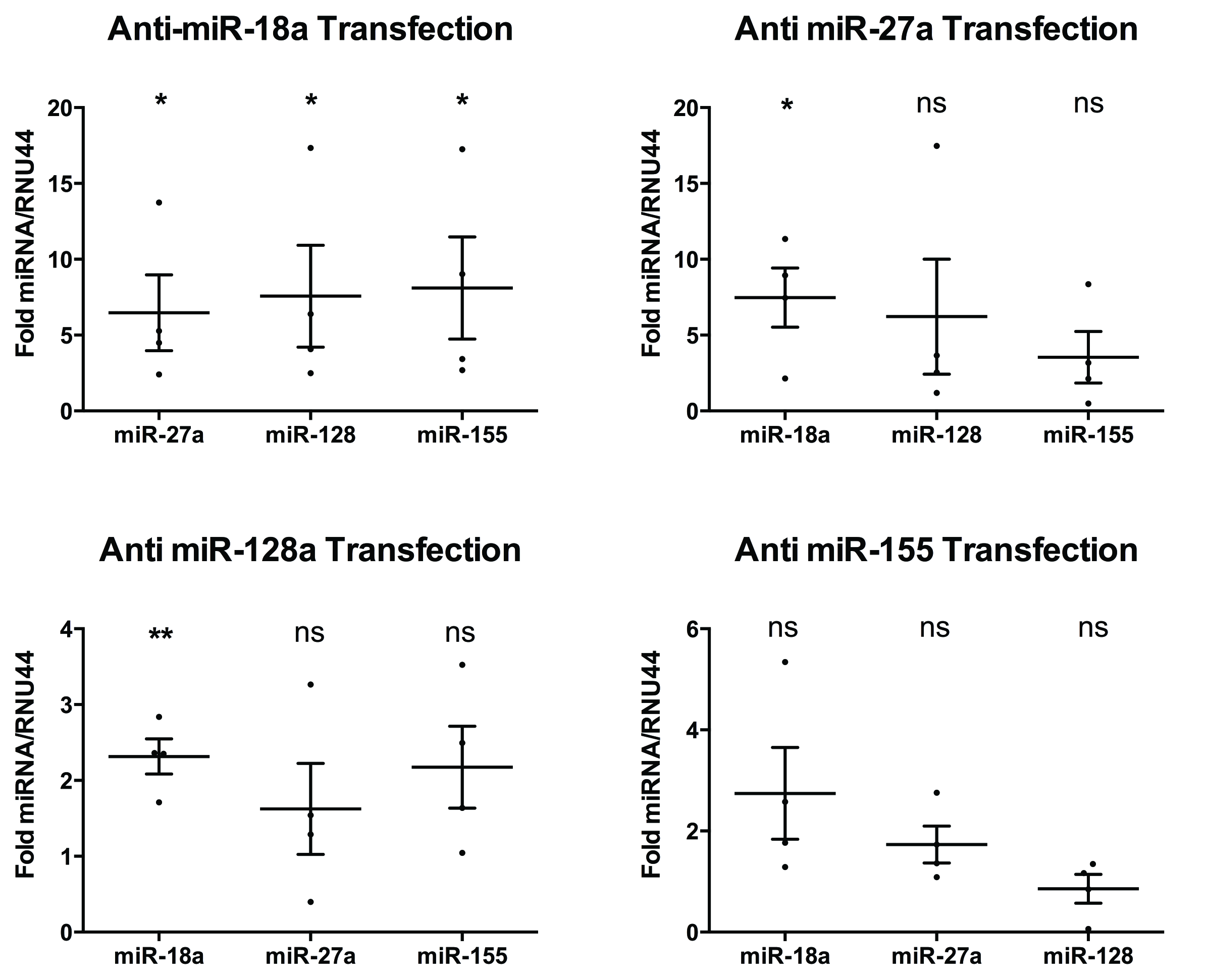

Supplement: Figure S5 — Effects of individual down-regulation of miR-18a, miR-27a, miR-128 and miR-155 on the expression levels of the candidate microRNAs. (TIF) [file pone.0111659.s005.tif]

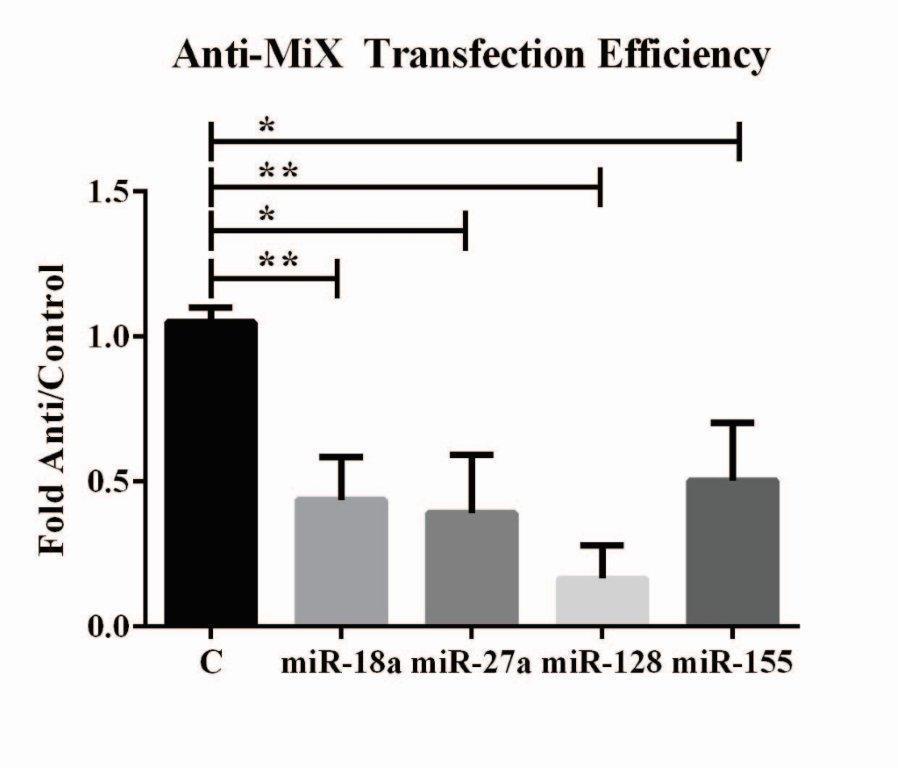

Supplement: Figure S6 — Transfection efficiency of pooled anti-miRs against miR-18a, miR-27a, miR-128 and miR-155. (JPG) [file pone.0111659.s006.jpg]

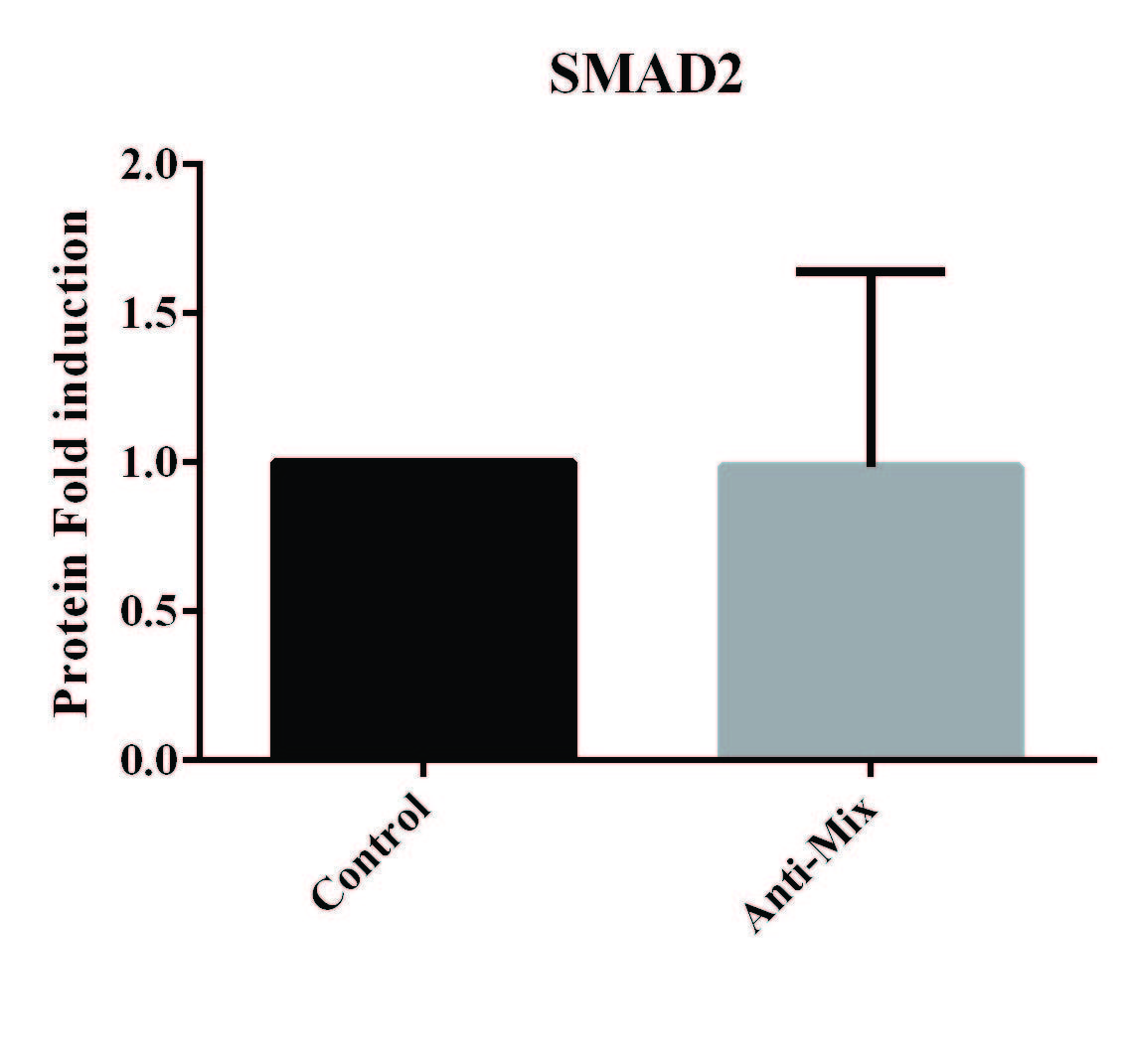

Supplement: Figure S7 — Effects of simultaneous down-regulation of miR-18a, miR-27a, miR-128 and miR-155 in SMAD2 protein levels. (JPG) [file pone.0111659.s007.jpg]
