# Supplementary material for: A MicroRNA Network Dysregulated in Asthma Controls IL-6 Production in Bronchial Epithelial Cells
Source: PLoS One. 2014 Oct 31;9(10):e111659. doi: 10.1371/journal.pone.0111659 (PMC4216117; doi:10.1371/journal.pone.0111659)
Supplement: Table S1 — Demographics of the population employed for microRNA profiling of human bronchial epithelial cells. (DOCX) [file pone.0111659.s008.docx]

| **Healthy** |  |  |  |  |  |
| --- | --- | --- | --- | --- | --- |
| **Volunteer ID** | **Age** | **Sex** | **% predicted FEV_1_** | **Array** | **Validation** |
| **HC01** | 24 | M | 103 |  | V |
| **HC02** | 27 | F | 83- |  | V |
| **HC03** | 21 | F | 117 | V | V |
| **HC04** | 24 | M | 83 | V | V |
| **HC05** | 20 | F | 101 | V | V |
| **HC06** | 20 | F | 96 |  | V |
| **HC07** | 20 | M | 98 | V | V |
| **HC08** | 22 | F | 119 |  | V |
| **HC09** | 20 | M | 102 |  | V |
| **HC10** | 33 | M | 105 |  | V |
| **HC11** | 27 | M | 110 |  | V |
| **HC12** | 22 | F | 126.7 | V | V |
| **HC13** | 21 | F | 90 |  | V |
| **Range** | **13** |  | **Average 104.2** |  |  |

| **Asthmatics** |  |  |  |  |  |
| --- | --- | --- | --- | --- | --- |
| **Patient ID** | **Age** | **Sex** | **% predicted FEV_1_** | Array | Validation |
| **A01** | 60 | M | 70 |  | V |
| **A02** | 44 | F | 82 |  | V |
| **A03** | 49 | F | 68 |  | V |
| **A04** | 56 | M | 70 | V | V |
| **A05** | 41 | F | 80 | V | V |
| **A06** | 47 | F | 91.4 | V | V |
| **A07** | 41 | F | 109 | V | V |
| **A08** | 64 | F | 69.3 |  | V |
| **A09** | 36 | F | 95.5 | V | V |
| **A10** | 42 | M | 68.7 |  | V |
| **A11** | 23 | M | 103 |  | V |
| **A12** | 52 | F | 102 |  | V |
| **A13** | 30 | F | 110 |  | V |
| **Syn D13** | 24 | F | 58 |  | V |
| **VK009c** | 23 | F | 87.8 |  | V |
| **Range** | **41** |  | **Average 85.5** |  |  |
