# Supplementary material for: A MicroRNA Network Dysregulated in Asthma Controls IL-6 Production in Bronchial Epithelial Cells
Source: PLoS One. 2014 Oct 31;9(10):e111659. doi: 10.1371/journal.pone.0111659 (PMC4216117; doi:10.1371/journal.pone.0111659)
Supplement: Table S2 — Bronchial epithelial cells microRNA array results. (DOCX) [file pone.0111659.s009.docx]

| **MicroRNA ID** | **Healthy Average** | **Asthma Average** |
| --- | --- | --- |
| **hsa-let-7a** | 1.2797086 | 1.0126812 |
| **hsa-let-7b** | 1.1700534 | 1.0554772 |
| **hsa-let-7c** | 1.0513696 | 1.006336 |
| **hsa-let-7d** | 1.7649796 | 1.0274648 |
| **hsa-let-7e** | 0.9126496 | 1.0080396 |
| **hsa-let-7f** | 2.109311 | 1.0602614 |
| **hsa-let-7g** | 1.2797698 | 1.0075262 |
| **hsa-miR-100** | 1.5966366 | 1.0318004 |
| **hsa-miR-101** | 6.8353886 | 1.3932356 |
| **hsa-miR-103** | 1.2982876 | 1.0068498 |
| **hsa-miR-106a** | 1.8055612 | 1.0055694 |
| **hsa-miR-106b** | 2.2131678 | 1.0095952 |
| **hsa-miR-107** | 18.5514776 | 1.4703776 |
| **hsa-miR-10a** | 2.5483606 | 1.0991536 |
| **hsa-miR-124** | 1.8779004 | 2.5479158 |
| **hsa-miR-125a-3p** | 0.5764916 | 6.1253502 |
| **hsa-miR-125a-5p** | 0.7783604 | 1.0068118 |
| **hsa-miR-125b** | 1.7163902 | 1.0443272 |
| **hsa-miR-126** | 1.1203354 | 1.0130014 |
| **hsa-miR-127-3p** | 8.9163832 | 4.3252524 |
| **hsa-miR-127-5p** | 0.9692716 | 1.0053634 |
| **hsa-miR-128** | 51.626679 | 1.0668068 |
| **hsa-miR-130a** | 1.3905354 | 1.0411838 |
| **hsa-miR-130b** | 1.7726894 | 1.0292982 |
| **hsa-miR-132** | 1.1695488 | 1.152926 |
| **hsa-miR-134** | 1.461904 | 1.7191176 |
| **hsa-miR-135a** | 3.2259874 | 1.0269996 |
| **hsa-miR-135b** | 1.7663802 | 1.011999 |
| **hsa-miR-138** | 1.1909122 | 1.1305684 |
| **hsa-miR-139-5p** | 1.157751 | 1.2778878 |
| **hsa-miR-140-3p** | 0.9727634 | 1.0358658 |
| **hsa-miR-140-5p** | 2.0207412 | 1.0127906 |
| **hsa-miR-141** | 1.802637 | 1.0113896 |
| **hsa-miR-146a** | 3.6532796 | 1.949507 |
| **hsa-miR-146b-5p** | 1.318201 | 1.0919506 |
| **hsa-miR-148a** | 2.5689124 | 1.0699894 |
| **hsa-miR-148b** | 2.2083352 | 1.0880186 |
| **hsa-miR-149** | 0.8122846 | 1.0709532 |
| **hsa-miR-152** | 1.4488258 | 1.043421 |
| **hsa-miR-155** | 1.8573296 | 1.006767 |
| **hsa-miR-15a** | 3.4404552 | 1.1712042 |
| **hsa-miR-15b** | 1.8987278 | 1.0645586 |
| **hsa-miR-16** | 1.7389502 | 1.015144 |
| **hsa-miR-17** | 1.9790666 | 1.0065618 |
| **hsa-miR-181a** | 1.190475 | 1.1010724 |
| **hsa-miR-181c** | 0.6380972 | 1.0784658 |
| **hsa-miR-182** | 0.9386436 | 1.0253288 |
| **hsa-miR-183** | 1.0102664 | 1.1227508 |
| **hsa-miR-185** | 1.8237564 | 1.0373914 |
| **hsa-miR-186** | 0.9717816 | 1.0212328 |
| **hsa-miR-18a** | 7.3940814 | 1.0411718 |
| **hsa-miR-18b** | 2.25359 | 1.3543486 |
| **hsa-miR-191** | 0.7808396 | 1.0205574 |
| **hsa-miR-192** | 1.2399858 | 1.0624306 |
| **hsa-miR-193a-3p** | 0.1072555 | 0.59468 |
| **hsa-miR-193a-5p** | 1.4013982 | 1.0227706 |
| **hsa-miR-193b** | 0.6759292 | 1.0685684 |
| **hsa-miR-194** | 1.2470196 | 1.0761766 |
| **hsa-miR-195** | 1.2552324 | 1.0183962 |
| **hsa-miR-197** | 1.1205536 | 1.1092506 |
| **hsa-miR-19a** | 2.1102124 | 1.0232888 |
| **hsa-miR-19b** | 2.0874054 | 1.0225 |
| **hsa-miR-200a** | 1.2982056 | 1.010597 |
| **hsa-miR-200b** | 1.1713598 | 1.0045844 |
| **hsa-miR-200c** | 0.94941 | 1.0094926 |
| **hsa-miR-202** | 0.415060333 | 1.810892 |
| **hsa-miR-203** | 1.8427792 | 1.2783878 |
| **hsa-miR-205** | 1.5220584 | 1.004692 |
| **hsa-miR-20a** | 2.1995506 | 1.012809 |
| **hsa-miR-20b** | 1.6455548 | 1.0181654 |
| **hsa-miR-21** | 2.9618376 | 1.0108654 |
| **hsa-miR-210** | 1.1203264 | 1.0204328 |
| **hsa-miR-212** | 0.9843712 | 1.9769432 |
| **hsa-miR-218** | 1.6796862 | 1.1713436 |
| **hsa-miR-22** | 3.362934 | 1.132804 |
| **hsa-miR-221** | 1.2040402 | 1.1266594 |
| **hsa-miR-222** | 0.7768208 | 1.0794604 |
| **hsa-miR-223** | 1.1060076 | 1.1632656 |
| **hsa-miR-224** | 2.2856196 | 1.0585908 |
| **hsa-miR-23a** | 3.0853842 | 1.1929558 |
| **hsa-miR-23b** | 6.1173174 | 1.0719592 |
| **hsa-miR-24** | 1.0289986 | 1.0212738 |
| **hsa-miR-25** | 2.7037934 | 1.0152784 |
| **hsa-miR-26a** | 1.3814754 | 1.010246 |
| **hsa-miR-26b** | 1.7869158 | 1.0032948 |
| **hsa-miR-27a** | 1.4773916 | 1.0227412 |
| **hsa-miR-27b** | 4.3230494 | 1.1211892 |
| **hsa-miR-28-3p** | 1.084591 | 1.0282658 |
| **hsa-miR-28-5p** | 1.628203 | 1.0419082 |
| **hsa-miR-296-5p** | 1.8719618 | 1.140743 |
| **hsa-miR-29a** | 1.036565 | 1.0066552 |
| **hsa-miR-29b** | 3.7855228 | 1.063076 |
| **hsa-miR-29c** | 0.7648128 | 1.0148632 |
| **hsa-miR-301a** | 6.4278484 | 1.0085988 |
| **hsa-miR-301b** | 38.959116 | 1.148039 |
| **hsa-miR-30b** | 1.1179374 | 1.00741 |
| **hsa-miR-30c** | 0.9639792 | 1.0051998 |
| **hsa-miR-31** | 1.0581952 | 1.0130962 |
| **hsa-miR-32** | 2.654664 | 1.4680616 |
| **hsa-miR-320** | 0.785535 | 1.0183976 |
| **hsa-miR-323-3p** | 0.5954524 | 1.088092 |
| **hsa-miR-324-3p** | 1.2658354 | 1.0683578 |
| **hsa-miR-324-5p** | 8.8504758 | 1.0102962 |
| **hsa-miR-328** | 1.9296428 | 1.311202 |
| **hsa-miR-330-3p** | 0.8761404 | 1.379118 |
| **hsa-miR-331-3p** | 1.1144706 | 1.0094618 |
| **hsa-miR-331-5p** | 15.7467106 | 1.2933624 |
| **hsa-miR-335** | 2.70912275 | 1.0123442 |
| **hsa-miR-339-3p** | 1.030203 | 1.1299766 |
| **hsa-miR-339-5p** | 1.7025772 | 1.094704 |
| **hsa-miR-340** | 2.8265976 | 1.005655 |
| **hsa-miR-342-3p** | 0.6999554 | 1.0520542 |
| **hsa-miR-345** | 2.0841452 | 1.0301956 |
| **hsa-miR-34a** | 3.0933918 | 1.0541382 |
| **hsa-miR-34c-5p** | 25.6732924 | 1.2613776 |
| **hsa-miR-361-5p** | 28.0504736 | 1.191144 |
| **hsa-miR-362-5p** | 1.1306264 | 1.031827 |
| **hsa-miR-365** | 0.9504488 | 1.069402 |
| **hsa-miR-374a** | 1.818188 | 1.0076386 |
| **hsa-miR-374b** | 1.5463552 | 1.0121538 |
| **hsa-miR-375** | 0.815009667 | 1.9279358 |
| **hsa-miR-376a** | 0.295628 | 0.546975 |
| **hsa-miR-376c** | 0.2895604 | 1.1561494 |
| **hsa-miR-410** | 0.164511 | 0.589753667 |
| **hsa-miR-411** | 0.1077958 | 2.9573212 |
| **hsa-miR-422a** | 0.6426128 | 1.0319582 |
| **hsa-miR-423-5p** | 5.1188832 | 1.6458142 |
| **hsa-miR-424** | 0.9851514 | 2.221724 |
| **hsa-miR-425** | 0.87346 | 1.0266952 |
| **hsa-miR-429** | 1.6558416 | 1.0015642 |
| **hsa-miR-449a** | 0.547033 | 0.66780125 |
| **hsa-miR-449b** | 5.9335658 | 2.4594722 |
| **hsa-miR-450b-5p** | 0.844053333 | 1.2490578 |
| **hsa-miR-452** | 1.9412504 | 1.0438148 |
| **hsa-miR-454** | 2.0919312 | 1.0126496 |
| **hsa-miR-455-3p** | 1.2487688 | 1.0369354 |
| **hsa-miR-455-5p** | 1.5089874 | 1.0208944 |
| **hsa-miR-483-5p** | 0.249185 | 1.3164072 |
| **hsa-miR-484** | 1.1779322 | 1.0070264 |
| **hsa-miR-485-3p** | 1.5644138 | 2.2449168 |
| **hsa-miR-486-5p** | 1.191334 | 0.78217525 |
| **hsa-miR-487a** | 1.4783118 | 0.437631 |
| **hsa-miR-489** | 1.8356334 | 1.130705 |
| **hsa-miR-491-5p** | 1.4785116 | 1.0359922 |
| **hsa-miR-494** | 1.918088 | 1.035483 |
| **hsa-miR-495** | 0.2630328 | 8.6707276 |
| **hsa-miR-500** | 3.2795486 | 1.1909134 |
| **hsa-miR-501-5p** | 0.87044 | 1.0642196 |
| **hsa-miR-502-3p** | 1.57683225 | 0.87219725 |
| **hsa-miR-503** | 0.4840318 | 1.07260875 |
| **hsa-miR-532-3p** | 1.112255 | 1.0087618 |
| **hsa-miR-532-5p** | 1.7293582 | 1.033376 |
| **hsa-miR-542-3p** | 0.97792275 | 0.316046333 |
| **hsa-miR-545** | 0.275085 | 1.931539 |
| **hsa-miR-574-3p** | 0.7232406 | 1.0548168 |
| **hsa-miR-582-5p** | 2.2386342 | 1.1763528 |
| **hsa-miR-590-5p** | 1.5213816 | 1.0039204 |
| **hsa-miR-598** | 2.979491 | 1.0158518 |
| **hsa-miR-618** | 0.8048114 | 1.7048466 |
| **hsa-miR-625** | 3.2288054 | 1.0244922 |
| **hsa-miR-628-5p** | 1.1010062 | 1.141242 |
| **hsa-miR-629** | 14.26827 | 1.5226928 |
| **hsa-miR-636** | 1.7381095 | 1.1967156 |
| **hsa-miR-642** | 0.631955 | 1.0974292 |
| **hsa-miR-652** | 3.3895048 | 1.090264 |
| **hsa-miR-660** | 2.2255278 | 1.020764 |
| **hsa-miR-671-3p** | 3.2501594 | 4.9279864 |
| **hsa-miR-708** | 0.9580594 | 1.0454238 |
| **hsa-miR-744** | 1.246865 | 1.0483538 |
| **hsa-miR-885-5p** | 0.158702 | 0.340446333 |
| **hsa-miR-886-3p** | 2.4681378 | 1.3070758 |
| **hsa-miR-886-5p** | 2.1389842 | 1.0905858 |
| **hsa-miR-92a** | 1.619631 | 1.0222468 |
| **hsa-miR-93** | 2.045322 | 1.0086236 |
| **hsa-miR-9** | 0.511132 | 0.6909675 |
| **hsa-miR-95** | 0.632232 | 0.565808 |
| **hsa-miR-96** | 1.0211562 | 2.6296598 |
| **hsa-miR-98** | 8.1734326 | 1.0670976 |
| **hsa-miR-99a** | 2.106695 | 1.0618618 |
| **hsa-miR-99b** | 1.452987 | 1.0094366 |
| **RNU48** | 1.108013 | 1.008763 |
| **U6** | 0.839315 | 1.0670516 |
