# Supplementary material for: A MicroRNA Network Dysregulated in Asthma Controls IL-6 Production in Bronchial Epithelial Cells
Source: PLoS One. 2014 Oct 31;9(10):e111659. doi: 10.1371/journal.pone.0111659 (PMC4216117; doi:10.1371/journal.pone.0111659)
Supplement: Table S3 — Candidate genes predicted to be targeted in each signalling pathway. (DOCX) [file pone.0111659.s010.docx]

**Pathways targeted by miR-18a, miR-27a, miR-128 and miR-155 and number of Targets**

| **TGFB** |  |  |  |  |  |  |
| --- | --- | --- | --- | --- | --- | --- |
| **MicroRNAs** | **Targets** |  | **MicroRNAs** | | **Targets** |  |
| hsa-miR-18a | 2 |  | hsa-miR-18a | | 3 |  |
| hsa-miR-27a | 15 |  | hsa-miR-27a | | 9 |  |
| hsa-miR-128 | 14 |  | hsa-miR-128 | | 8 |  |
| hsa-miR-155 | 5 |  | hsa-miR-155 | | 5 |  |
| **Overall number of Targets: 21** | |  | **Overall number of Targets: 17** | | |  |
|  |  |  |  |  | |  |
|  |  |  |  |  | |  |
| **MAPK** |  |  |  |  |  |  |
| **MicroRNAs** | **Targets** |  | **MicroRNAs** | | **Targets** |  |
| hsa-miR-18a | 7 |  | hsa-miR-18a | | 3 |  |
| hsa-miR-27a | 27 |  | hsa-miR-27a | | 17 |  |
| hsa-miR-128 | 24 |  | hsa-miR-128 | | 16 |  |
| hsa-miR-155 | 13 |  | hsa-miR-155 | | 4 |  |
| **Overall number of Targets: 43** | |  | **Overall number of Targets: 25** | | |  |
|  |  |  |  |  | |  |
| **mTOR** |  |  |  |  | |  |
| **List names** | **number of elements** |  |  |  | |  |
| hsa-miR-18a | 2 |  |  |  | |  |
| hsa-miR-27a | 5 |  |  |  | |  |
| hsa-miR-128 | 7 |  |  |  | |  |
| hsa-miR-155 | 7 |  |  |  | |  |
| **Overall number of Targets: 14** | |  |  |  | |  |
|  |  |  |  |  | |  |

**Targets within each pathway: microRNA and Gene IDs**

| **TGFB** |  |  |
| --- | --- | --- |
| **MicroRNAs** | **No. Genes** | **Gene IDs** |
| hsa-miR-18a hsa-miR-27a hsa-miR-128 hsa-miR-155 | 1 | SMAD2 |
| hsa-miR-27a hsa-miR-128 hsa-miR-155 | 1 | RPS6KB1 |
| hsa-miR-27a hsa-miR-128 | 10 | BMPR2 |
|  |  | SMAD9 |
|  |  | IFNG |
|  |  | SMAD5 |
|  |  | GDF6 |
|  |  | ACVR2A |
|  |  | TGFBR1 |
|  |  | SP1 |
|  |  | ID3 |
|  |  | SMURF2 |
| hsa-miR-18a | 1 | THBS1 |
| hsa-miR-27a | 3 | CHRD |
|  |  | ID4 |
|  |  | BMPR1A |
| hsa-miR-128 | 2 | LTBP1 |
|  |  | SMAD4 |
| hsa-miR-155 | 3 | SMAD1 |
|  |  | PPP2CB |
|  |  | ACVR1 |

| **MAPK** |  |  |
| --- | --- | --- |
| **MicroRNAs** | **No. Genes** | **Gene IDs** |
| hsa-miR-18a hsa-miR-27a hsa-miR-128 | 1 | CDC42 |
| hsa-miR-27a hsa-miR-128 hsa-miR-155 | 2 | SOS1 |
|  |  | RAP1B |
| hsa-miR-18a hsa-miR-27a | 2 | MEF2C |
|  |  | DUSP16 |
| hsa-miR-27a hsa-miR-128 | 18 | CDC25B |
|  |  | MKNK2 |
|  |  | CACNB2 |
|  |  | FGF1 |
|  |  | MAP3K4 |
|  |  | TGFBR1 |
|  |  | PDGFRA |
|  |  | EVI1 |
|  |  | MAPKAPK3 |
|  |  | MAPK14 |
|  |  | MAP2K7 |
|  |  | DUSP5 |
|  |  | PRKY |
|  |  | MAP2K4 |
|  |  | PRKX |
|  |  | RPS6KA5 |
|  |  | NF1 |
|  |  | GRB2 |
| hsa-miR-27a hsa-miR-155 | 2 | KRAS |
|  |  | MAP3K14 |
| hsa-miR-18a | 4 | TAOK3 |
|  |  | MAP3K1 |
|  |  | CACNB3 |
|  |  | ENSG00000091436 |
| hsa-miR-27a | 2 | MAP3K12 |
|  |  | NLK |
| hsa-miR-128 | 3 | EGFR |
|  |  | PTPN5 |
|  |  | CRKL |
| hsa-miR-155 | 9 | BDNF |
|  |  | FGF7 |
|  |  | MAP4K3 |
|  |  | FOS |
|  |  | RPS6KA3 |
|  |  | MAP3K10 |
|  |  | MAP3K7IP2 |
|  |  | ARRB2 |
|  |  | DUSP14 |

| **mTOR** |  |  |
| --- | --- | --- |
| **MicroRNAs** | **No. Genes** | **Gene IDs** |
| hsa-miR-27a hsa-miR-128 hsa-miR-155 | 1 | RPS6KB1 |
| hsa-miR-18a hsa-miR-155 | 1 | HIF1A |
| hsa-miR-27a hsa-miR-128 | 4 | VEGFB |
|  |  | TSC1 |
|  |  | VEGFC |
|  |  | PDPK1 |
| hsa-miR-18a | 1 | IGF1 |
| hsa-miR-128 | 2 | ULK1 |
|  |  | PIK3R1 |
| hsa-miR-155 | 5 | CAB39 |
|  |  | ULK2 |
|  |  | RHEB |
|  |  | RPS6KA3 |
|  |  | ENSG00000164327 |

| **Jak-STAT signalling** |  |  |
| --- | --- | --- |
| **MicroRNAs** | **No. Genes** | **Gene IDs** |
| hsa-miR-27a hsa-miR-128 hsa-miR-155 | 1 | SOS1 |
| hsa-miR-18a hsa-miR-128 | 1 | SOCS5 |
| hsa-miR-27a hsa-miR-128 | 5 | IFNG |
|  |  | LIFR |
|  |  | LEP |
|  |  | SPRY2 |
|  |  | GRB2 |
| hsa-miR-18a | 2 | CCND2 |
|  |  | STAM2 |
| hsa-miR-27a | 3 | SOCS4 |
|  |  | CBLB |
|  |  | IL10 |
| hsa-miR-128 | 1 | PIK3R1 |
| hsa-miR-155 | 4 | IL6ST |
|  |  | SPRED1 |
|  |  | SOCS1 |
|  |  | CBL |

| **Cytokine-cytokine receptor interaction** | |  |
| --- | --- | --- |
| **Names** | **total** | **elements** |
| hsa-miR-27a hsa-miR-128 | 15 | VEGFC |
|  |  | NGFR |
|  |  | BMPR2 |
|  |  | CXCL2 |
|  |  | IFNG |
|  |  | EDAR |
|  |  | CSF1 |
|  |  | VEGFB |
|  |  | LIFR |
|  |  | ACVR2A |
|  |  | TGFBR1 |
|  |  | LEP |
|  |  | PDGFRA |
|  |  | MET |
|  |  | KITLG |
| hsa-miR-18a | 3 | KIT |
|  |  | PDGFC |
|  |  | LTBR |
| hsa-miR-27a | 2 | IL10 |
|  |  | BMPR1A |
| hsa-miR-128 | 1 | EGFR |
| hsa-miR-155 | 4 | IL6ST |
|  |  | CSF1R |
|  |  | IL1RAP |
|  |  | ACVR1 |
