# Supplementary material for: A MicroRNA Network Dysregulated in Asthma Controls IL-6 Production in Bronchial Epithelial Cells
Source: PLoS One. 2014 Oct 31;9(10):e111659. doi: 10.1371/journal.pone.0111659 (PMC4216117; doi:10.1371/journal.pone.0111659)
Supplement: Table S4 — Candidate genes predicted to be targeted in TGF-β and IFNs signalling pathway as well as IL-6 and IL-8 secretion pathways. (DOCX) [file pone.0111659.s011.docx]

| **IFN** | |
| --- | --- |
| **MicroRNA** | **Gene ID** |
| miR-18a | IRF2 |
| miR-18a | IFNA |
| miR-18a | MEKK1 |
| miR-18a | MSK1 |
| miR-18a | IFNAR2 |
| miR-27a | RPS6KB1 |
| miR-27a | MSK1 |
| miR-27a | IFNAR1 |
| miR-27a | MSK2 |
| miR-27a | IRF5 |
| miR-27a | CREB1 |
| miR-128 | IFNA |
| miR-128 | RPS6KB1 |
| miR-128 | MSK1 |
| miR-155 | SOCS1 |
| miR-155 | STAT1 |
| miR-155 | PIK3R1 |
| miR-155 | RPS6KB1 |
| miR-155 | IRF8 |
| miR-155 | RAC1 |

| **TGF-β** | |
| --- | --- |
| **MicroRNA** | **Gene ID** |
| miR-18a | SMAD2 |
| miR-18a | SMAD3 |
| miR-18a | SMAD4 |
| miR-27a | SMAD2 |
| miR-27a | SMAD4 |
| miR-27a | SMURF2 |
| miR-128 | SMAD2 |
| miR-128 | SMAD4 |
| miR-128 | SMURF2 |
| miR-128 | TGFBR1 |
| miR-155 | SMAD2 |
| miR-155 | SMAD3 |
| miR-155 | SMAD6 |
| miR-155 | SMURF2 |
| miR-155 | TGFBR2 |

| **IL-6 secretion** | |
| --- | --- |
| **MicroRNA** | **Gene ID** |
| miR-18a | SOS1 |
| miR-18a | IKBKE |
| miR-18a | NFKBIZ |
| miR-27a | IL6ST |
| miR-27a | NFKBID |
| miR-27a | SOS1 |
| miR-27a | RPS6KB1 |
| miR-128 | SOS1 |
| miR-128 | RPS6KB1 |
| miR-155 | NFKB |
| miR-155 | IKBIP |
| miR-155 | STAT1 |
| miR-155 | JAK2 |
| miR-155 | PIK3R1 |
| miR-155 | RPS6KB1 |
| miR-155 | IKBKE |
| miR-155 | FOS |
| miR-155 | CEBPB |

| **IL-8 secretion** | |
| --- | --- |
| **microRNA** | **Gene ID** |
| miR-18a | IKBKE |
| miR-18a | NFKBIZ |
| miR-27a | NFKBID |
| miR-27a | RPS6KB1 |
| miR-128 | RPS6KB1 |
| miR-155 | RPS6KB1 |
| miR-155 | JAK2 |
| miR-155 | NFKB |
| miR-155 | PIK3R1 |
| miR-155 | IKBKE |
| miR-155 | IKBIP |
| miR-155 | FOS |
