# Supplementary material for: A MicroRNA Network Dysregulated in Asthma Controls IL-6 Production in Bronchial Epithelial Cells
Source: PLoS One. 2014 Oct 31;9(10):e111659. doi: 10.1371/journal.pone.0111659 (PMC4216117; doi:10.1371/journal.pone.0111659)
Supplement: Table S5 — Complete set of candidate genes predicted to be targeted by miR-18a, miR-27a, miR-128 and miR-155. (DOCX) [file pone.0111659.s012.docx]

| **MicroRNAs** | **No. Genes** | **Gene ID** |
| --- | --- | --- |
| miR-18a miR-27a miR-128 miR-155 | 6 | MBNL2 |
|  |  | KCNA1 |
|  |  | QKI |
|  |  | SMAD2 |
|  |  | PLAG1 |
|  |  | ATP2B1 |
| miR-18a miR-27a miR-128 | 14 | NEDD4 |
|  |  | C5orf13 |
|  |  | C7orf42 |
|  |  | PAPPA |
|  |  | RUNX1 |
|  |  | EPB41L1 |
|  |  | KIAA1012 |
|  |  | NAT13 |
|  |  | C1orf9 |
|  |  | PRICKLE2 |
|  |  | DCUN1D4 |
|  |  | HCN4 |
|  |  | SH3RF1 |
|  |  | KPNA6 |
| miR-18a miR-27a miR-155 | 1 | RAB11FIP2 |
| miR-27a miR-128 miR-155 | 28 | RXRA |
|  |  | SOCS6 |
|  |  | MIER3 |
|  |  | CAMTA1 |
|  |  | FBXO33 |
|  |  | SERTAD2 |
|  |  | EYA1 |
|  |  | ZFHX4 |
|  |  | PANK1 |
|  |  | APAF1 |
|  |  | TMED5 |
|  |  | NR1D2 |
|  |  | WEE1 |
|  |  | NFIB |
|  |  | BCORL1 |
|  |  | SP1 |
|  |  | FAM134C |
|  |  | PRKY |
|  |  | CAB39 |
|  |  | PKIA |
|  |  | JMJD1A |
|  |  | N4BP1 |
|  |  | NOVA1 |
|  |  | TAPT1 |
|  |  | SOX11 |
|  |  | ELL2 |
|  |  | C5orf41 |
|  |  | TMTC2 |
| miR-18a miR-27a | 14 | IGF1 |
|  |  | ALCAM |
|  |  | HDHD2 |
|  |  | PHLPPL |
|  |  | SETD8 |
|  |  | PFTK1 |
|  |  | TNRC6B |
|  |  | FBXO34 |
|  |  | C1orf173 |
|  |  | NAV1 |
|  |  | TARDBP |
|  |  | ENC1 |
|  |  | HMGCS1 |
|  |  | PARD6B |
| miR-18a miR-128 | 11 | FRYL |
|  |  | TBC1D9B |
|  |  | LOC399947 |
|  |  | NEUROD1 |
|  |  | FRMD4A |
|  |  | LIN28 |
|  |  | NCOA1 |
|  |  | CTDSPL |
|  |  | UBE2Z |
|  |  | WDR68 |
|  |  | CCDC88A |
| miR-18a miR-155 | 19 | ADD3 |
|  |  | MECP2 |
|  |  | CLCC1 |
|  |  | TRIM2 |
|  |  | SLC12A6 |
|  |  | RABGAP1 |
|  |  | REPS2 |
|  |  | NFAT5 |
|  |  | VPS13A |
|  |  | JARID1B |
|  |  | ODZ3 |
|  |  | TSHZ3 |
|  |  | HIF1A |
|  |  | GABRA4 |
|  |  | KLHDC5 |
|  |  | SIM2 |
|  |  | SOCS5 |
|  |  | ARL15 |
|  |  | SATB1 |
| miR-27a miR-128 | 380 | C2orf55 |
|  |  | C15orf27 |
|  |  | ITGA5 |
|  |  | BTG2 |
|  |  | RNF38 |
|  |  | GLTP |
|  |  | TMSB10 |
|  |  | RBJ |
|  |  | CYP39A1 |
|  |  | GALNT3 |
|  |  | EPB41L4A |
|  |  | KCNA6 |
|  |  | KBTBD8 |
|  |  | HMGB3 |
|  |  | GOLM1 |
|  |  | MED13L |
|  |  | KIAA1199 |
|  |  | HOXA13 |
|  |  | DKK2 |
|  |  | RPS6KA5 |
|  |  | ATXN10 |
|  |  | C10orf56 |
|  |  | KIAA1033 |
|  |  | PSMA1 |
|  |  | C12orf34 |
|  |  | ISL1 |
|  |  | SFRS1 |
|  |  | KPNA3 |
|  |  | TBX5 |
|  |  | UBE2F |
|  |  | GFPT2 |
|  |  | DVL2 |
|  |  | ZNF148 |
|  |  | KIAA2018 |
|  |  | IKZF1 |
|  |  | CTDSP2 |
|  |  | CHST1 |
|  |  | ING5 |
|  |  | GRB2 |
|  |  | PRDM16 |
|  |  | HBEGF |
|  |  | PAK6 |
|  |  | ABCA1 |
|  |  | CSF1 |
|  |  | FAM105B |
|  |  | TXNDC1 |
|  |  | MEIS2 |
|  |  | CPEB3 |
|  |  | SLC6A6 |
|  |  | DUSP5 |
|  |  | NDUFS4 |
|  |  | CDS1 |
|  |  | ADAM19 |
|  |  | FAM133B |
|  |  | USP42 |
|  |  | EDNRA |
|  |  | IRS1 |
|  |  | PIB5PA |
|  |  | APPBP2 |
|  |  | RCOR3 |
|  |  | NRIP1 |
|  |  | NRP2 |
|  |  | H3F3B |
|  |  | NCAM1 |
|  |  | SLC38A4 |
|  |  | CASC3 |
|  |  | PALM2 |
|  |  | LSM12 |
|  |  | ITSN2 |
|  |  | RCAN2 |
|  |  | GNG12 |
|  |  | C17orf85 |
|  |  | PPARG |
|  |  | ARMC8 |
|  |  | RNF144A |
|  |  | EVI5 |
|  |  | STK40 |
|  |  | MED14 |
|  |  | EFHA2 |
|  |  | SPRY2 |
|  |  | RET |
|  |  | STBD1 |
|  |  | C1orf21 |
|  |  | LITAF |
|  |  | UBE2W |
|  |  | CDH24 |
|  |  | CNR1 |
|  |  | FAM126B |
|  |  | MYT1 |
|  |  | MSI1 |
|  |  | UBE2N |
|  |  | NFE2L2 |
|  |  | NEK6 |
|  |  | ACVR2A |
|  |  | PGM2L1 |
|  |  | OPA1 |
|  |  | PPP1CC |
|  |  | WNT3A |
|  |  | TMEM25 |
|  |  | DCX |
|  |  | UBR5 |
|  |  | MAPK14 |
|  |  | NPTX2 |
|  |  | RORB |
|  |  | SLC35F1 |
|  |  | SATB2 |
|  |  | KIAA1787 |
|  |  | HOXB8 |
|  |  | SNAP25 |
|  |  | FZD7 |
|  |  | ADCY3 |
|  |  | VANGL2 |
|  |  | APBA2 |
|  |  | AFF4 |
|  |  | SAMD10 |
|  |  | NGFR |
|  |  | NGFRAP1 |
|  |  | EDAR |
|  |  | C6orf60 |
|  |  | NHLH2 |
|  |  | ANK2 |
|  |  | SLC1A2 |
|  |  | CPD |
|  |  | MEPCE |
|  |  | HOXA10 |
|  |  | BRSK1 |
|  |  | C1orf34 |
|  |  | ZNF827 |
|  |  | RNF12 |
|  |  | MFSD2 |
|  |  | SRGAP2 |
|  |  | TMEM9B |
|  |  | AQP11 |
|  |  | SH3BGRL2 |
|  |  | MATN3 |
|  |  | GNS |
|  |  | EBF3 |
|  |  | NF1 |
|  |  | LYPD3 |
|  |  | NAV2 |
|  |  | TGFBR1 |
|  |  | FAM78A |
|  |  | CCNK |
|  |  | CNOT7 |
|  |  | C1orf52 |
|  |  | PDS5B |
|  |  | PTER |
|  |  | SFRS2IP |
|  |  | ARHGAP12 |
|  |  | C7orf41 |
|  |  | DPY19L3 |
|  |  | ALG9 |
|  |  | GALNT7 |
|  |  | CABLES2 |
|  |  | MAN2A1 |
|  |  | SH2D3C |
|  |  | RND3 |
|  |  | PTPRT |
|  |  | PDE3A |
|  |  | FRS3 |
|  |  | MAP2K4 |
|  |  | WSB1 |
|  |  | NXF1 |
|  |  | PCTK3 |
|  |  | SGMS1 |
|  |  | PLEKHH2 |
|  |  | MANEAL |
|  |  | CDH11 |
|  |  | SLC7A11 |
|  |  | PPM1E |
|  |  | SHANK3 |
|  |  | GREM1 |
|  |  | AK2 |
|  |  | SPATA2 |
|  |  | NRXN1 |
|  |  | UNKL |
|  |  | GNB2 |
|  |  | POU3F2 |
|  |  | PTPN9 |
|  |  | ARRDC4 |
|  |  | E2F7 |
|  |  | MDFI |
|  |  | C10orf114 |
|  |  | FOXO1 |
|  |  | ST6GALNAC3 |
|  |  | CA12 |
|  |  | DOT1L |
|  |  | SEC61A1 |
|  |  | DLL4 |
|  |  | SEMA6A |
|  |  | STK39 |
|  |  | PTGER4 |
|  |  | SMARCA1 |
|  |  | RGS1 |
|  |  | PLXND1 |
|  |  | PDE7B |
|  |  | YWHAB |
|  |  | PDIA5 |
|  |  | ZDHHC17 |
|  |  | IRF4 |
|  |  | KIAA1737 |
|  |  | FOXP4 |
|  |  | UBR1 |
|  |  | KCNK2 |
|  |  | WDTC1 |
|  |  | UBE2NL |
|  |  | SYT1 |
|  |  | FAM126A |
|  |  | FOXP2 |
|  |  | EPB41 |
|  |  | RARA |
|  |  | MKNK2 |
|  |  | RMND5A |
|  |  | MARCKS |
|  |  | PPARA |
|  |  | MOSPD3 |
|  |  | CAPZA1 |
|  |  | PLK2 |
|  |  | MOBKL1A |
|  |  | NFASC |
|  |  | ELFN2 |
|  |  | FOXN3 |
|  |  | MIER2 |
|  |  | AGRN |
|  |  | NPEPPS |
|  |  | P2RY5 |
|  |  | TSC1 |
|  |  | MESDC1 |
|  |  | RAP1B |
|  |  | OTX2 |
|  |  | SLC6A1 |
|  |  | USP46 |
|  |  | CCNJ |
|  |  | STIM2 |
|  |  | SPTY2D1 |
|  |  | RPGRIP1L |
|  |  | HNRNPF |
|  |  | EGFR |
|  |  | TNPO1 |
|  |  | ACOT11 |
|  |  | AFAP1 |
|  |  | PGAP1 |
|  |  | PPME1 |
|  |  | CHD2 |
|  |  | CREB1 |
|  |  | ABCB9 |
|  |  | NEO1 |
|  |  | STAG1 |
|  |  | CD28 |
|  |  | CCNG1 |
|  |  | AMMECR1L |
|  |  | SLC39A13 |
|  |  | C10orf137 |
|  |  | TROVE2 |
|  |  | CA10 |
|  |  | NR5A2 |
|  |  | EHD3 |
|  |  | C1orf108 |
|  |  | CPEB4 |
|  |  | ACVR1 |
|  |  | PLEKHH1 |
|  |  | BAG2 |
|  |  | MAP2K7 |
|  |  | ATPAF1 |
|  |  | PLAGL2 |
|  |  | TMCC1 |
|  |  | ELMO1 |
|  |  | ANK1 |
|  |  | DCP2 |
|  |  | SEMA6D |
|  |  | GSPT1 |
|  |  | ADCY6 |
|  |  | ID2 |
|  |  | TMEM189-UBE2V1 |
|  |  | LOC440093 |
|  |  | BAHD1 |
|  |  | FLRT2 |
|  |  | PPP4C |
|  |  | CACNB2 |
|  |  | EN2 |
|  |  | ST14 |
|  |  | FBXW7 |
|  |  | CLCN3 |
|  |  | SFRS12 |
|  |  | GPAM |
|  |  | MAP3K7IP3 |
|  |  | JMJD1C |
|  |  | RAB11FIP1 |
|  |  | ADORA2B |
|  |  | NR2F6 |
|  |  | CADM1 |
|  |  | TMUB1 |
|  |  | EIF2C2 |
|  |  | KTELC1 |
|  |  | FLRT3 |
|  |  | B4GALT3 |
|  |  | SYDE1 |
|  |  | NHS |
|  |  | RNF139 |
|  |  | MNT |
|  |  | INSR |
|  |  | GLT25D2 |
|  |  | RGS6 |
|  |  | SLITRK1 |
|  |  | CIT |
|  |  | MMD |
|  |  | NEUROD6 |
|  |  | UNC5D |
|  |  | SLC39A11 |
|  |  | ARFGEF1 |
|  |  | GPD2 |
|  |  | ZNF800 |
|  |  | UNC13C |
|  |  | GATA2 |
|  |  | SV2A |
|  |  | PDGFRA |
|  |  | MAPK8IP3 |
|  |  | E2F6 |
|  |  | ZFHX3 |
|  |  | C1orf144 |
|  |  | EYA4 |
|  |  | BMI1 |
|  |  | ITPKC |
|  |  | EIF2S2 |
|  |  | ZHX1 |
|  |  | SS18L1 |
|  |  | FOSB |
|  |  | BCL3 |
|  |  | UBE2V1 |
|  |  | SLCO5A1 |
|  |  | NARG1 |
|  |  | TLK2 |
|  |  | ZZZ3 |
|  |  | ZNF385A |
|  |  | C16orf5 |
|  |  | PAQR9 |
|  |  | STX16 |
|  |  | HOXC6 |
|  |  | PHB |
|  |  | KPNB1 |
|  |  | NRBF2 |
|  |  | CCDC92 |
|  |  | NCOA7 |
|  |  | LONRF1 |
|  |  | NXT2 |
|  |  | FAM108B1 |
|  |  | HIP1 |
|  |  | LIMK1 |
|  |  | SEC24A |
|  |  | RELN |
|  |  | NRK |
|  |  | ONECUT2 |
|  |  | POM121 |
|  |  | LBH |
|  |  | EPHB2 |
|  |  | RUNDC3A |
|  |  | UBE2Q1 |
|  |  | CCDC71 |
|  |  | ANKRD43 |
|  |  | LPCAT1 |
|  |  | DTNA |
|  |  | SFRP1 |
|  |  | CDH5 |
|  |  | EDEM3 |
|  |  | TMEM110 |
|  |  | NEK2 |
|  |  | YPEL3 |
|  |  | ORC5L |
|  |  | VEGFB |
|  |  | HLX |
|  |  | DNAJC13 |
|  |  | RYBP |
|  |  | PNKD |
|  |  | AXUD1 |
|  |  | HAPLN1 |
|  |  | ProSAPiP1 |
|  |  | ZADH2 |
|  |  | SGPP1 |
|  |  | CBFA2T3 |
|  |  | RNGTT |
| miR-27a miR-155 | 31 | RTKN2 |
|  |  | TRIM23 |
|  |  | EXOC5 |
|  |  | MRPS14 |
|  |  | APOL6 |
|  |  | MAP3K14 |
|  |  | C8orf4 |
|  |  | KRAS |
|  |  | ANTXR2 |
|  |  | HDAC9 |
|  |  | GPR126 |
|  |  | LIN28B |
|  |  | ELAVL2 |
|  |  | PCDHA9 |
|  |  | ZBTB41 |
|  |  | C6orf120 |
|  |  | FBXO30 |
|  |  | NR2F2 |
|  |  | ZIC5 |
|  |  | MAP3K7IP2 |
|  |  | HIVEP2 |
|  |  | CHD7 |
|  |  | ZNF238 |
|  |  | SCAMP1 |
|  |  | RNF111 |
|  |  | PSKH1 |
|  |  | SLFN11 |
|  |  | KIF3A |
|  |  | APC |
|  |  | VAV3 |
|  |  | GOLT1B |
| miR-128 miR-155 | 12 | CTDSPL2 |
|  |  | COL21A1 |
|  |  | KLHL4 |
|  |  | MYBL1 |
|  |  | F13A1 |
|  |  | FAR1 |
|  |  | WTAP |
|  |  | NAV3 |
|  |  | BCL11A |
|  |  | ZNF192 |
|  |  | ZNF652 |
|  |  | RPS6KB1 |
| miR-18a | 162 | KCNMA1 |
|  |  | KCNJ2 |
|  |  | MEF2D |
|  |  | UBE2G1 |
|  |  | GUCY1A3 |
|  |  | CA13 |
|  |  | SH3BP4 |
|  |  | AP3S2 |
|  |  | TAOK3 |
|  |  | HNMT |
|  |  | DIP2C |
|  |  | SOX21 |
|  |  | ACSL3 |
|  |  | ZNF704 |
|  |  | ZIC2 |
|  |  | FAM73A |
|  |  | DICER1 |
|  |  | PIAS3 |
|  |  | ST8SIA4 |
|  |  | ORAI3 |
|  |  | SON |
|  |  | SEC23IP |
|  |  | HSF2 |
|  |  | RNF145 |
|  |  | FAM130A2 |
|  |  | SULT1C2 |
|  |  | AEBP2 |
|  |  | VTCN1 |
|  |  | LTBR |
|  |  | GRHL2 |
|  |  | COQ10B |
|  |  | HEXIM1 |
|  |  | NR3C1 |
|  |  | PARP11 |
|  |  | ZC3H6 |
|  |  | ASXL2 |
|  |  | DDX42 |
|  |  | TLL2 |
|  |  | YPEL5 |
|  |  | SDC4 |
|  |  | NKIRAS1 |
|  |  | TEX2 |
|  |  | NIP30 |
|  |  | CRIM1 |
|  |  | ATM |
|  |  | ESR1 |
|  |  | ZBTB47 |
|  |  | HMBOX1 |
|  |  | PDE4D |
|  |  | TRPC4 |
|  |  | PRKACB |
|  |  | DSC1 |
|  |  | UQCRQ |
|  |  | XYLT2 |
|  |  | FCHSD2 |
|  |  | DOCK4 |
|  |  | CAMK2N1 |
|  |  | SAR1A |
|  |  | RFC4 |
|  |  | GAB2 |
|  |  | ZBTB4 |
|  |  | TNFAIP3 |
|  |  | SNURF |
|  |  | CLASP2 |
|  |  | LRRFIP1 |
|  |  | GCLC |
|  |  | MAN1A2 |
|  |  | FKTN |
|  |  | CTGF |
|  |  | KIF3B |
|  |  | TOR1B |
|  |  | CREBL2 |
|  |  | DAAM2 |
|  |  | FAM3C |
|  |  | MDGA1 |
|  |  | BHLHB5 |
|  |  | IGF2BP2 |
|  |  | C20orf121 |
|  |  | BTN1A1 |
|  |  | ZNF365 |
|  |  | FAM8A1 |
|  |  | USP6 |
|  |  | KIAA0513 |
|  |  | FAM136A |
|  |  | PACSIN1 |
|  |  | ANKRD13C |
|  |  | MBNL1 |
|  |  | ZHX2 |
|  |  | CAD |
|  |  | AKR1D1 |
|  |  | CENTA2 |
|  |  | ZNF501 |
|  |  | RBBP8 |
|  |  | ANKRD50 |
|  |  | THBS1 |
|  |  | PHF2 |
|  |  | RNASEL |
|  |  | STK4 |
|  |  | CAMSAP1L1 |
|  |  | ZNF367 |
|  |  | LYCAT |
|  |  | TRIOBP |
|  |  | C5orf30 |
|  |  | EHMT1 |
|  |  | CDC2L6 |
|  |  | PDZD2 |
|  |  | ERLIN1 |
|  |  | DZIP3 |
|  |  | RAB5C |
|  |  | MESP1 |
|  |  | IRF2 |
|  |  | CHRM2 |
|  |  | IDH3G |
|  |  | OLFML2B |
|  |  | OPRM1 |
|  |  | UBTD2 |
|  |  | VPS54 |
|  |  | BRWD3 |
|  |  | MSL2L1 |
|  |  | CLIP3 |
|  |  | PURB |
|  |  | GLRB |
|  |  | SRGAP3 |
|  |  | C20orf30 |
|  |  | TGFBR3 |
|  |  | PTGFRN |
|  |  | FOXN1 |
|  |  | PHF20L1 |
|  |  | ATXN1 |
|  |  | NEDD9 |
|  |  | FBXL11 |
|  |  | KLHL20 |
|  |  | CAMKK2 |
|  |  | RAB9A |
|  |  | JUB |
|  |  | DUSP16 |
|  |  | POLR3F |
|  |  | TMEM2 |
|  |  | NR1I2 |
|  |  | INADL |
|  |  | ETV6 |
|  |  | LOC203547 |
|  |  | REXO2 |
|  |  | CYP4F11 |
|  |  | NRG1 |
|  |  | GAB1 |
|  |  | CEP57 |
|  |  | MAPK4 |
|  |  | SMAP2 |
|  |  | MAP7D1 |
|  |  | DPP10 |
|  |  | GIGYF1 |
|  |  | C20orf59 |
|  |  | TRIB2 |
|  |  | INPP5A |
|  |  | BTG3 |
|  |  | BRWD1 |
|  |  | TRADD |
|  |  | PSD3 |
|  |  | DCLRE1C |
|  |  | FNBP1 |
|  |  | SLC35B4 |
| miR-27a | 426 | PTGER3 |
|  |  | CD44 |
|  |  | PKNOX2 |
|  |  | C1orf106 |
|  |  | ATRX |
|  |  | ANKRD36B |
|  |  | MOSC1 |
|  |  | PELI2 |
|  |  | CCDC46 |
|  |  | MITF |
|  |  | SENP1 |
|  |  | KRT82 |
|  |  | MMP13 |
|  |  | CAPN3 |
|  |  | C17orf63 |
|  |  | ERG |
|  |  | AP1G1 |
|  |  | DIRAS1 |
|  |  | GDA |
|  |  | RAB3IP |
|  |  | ZNF230 |
|  |  | GK |
|  |  | SERINC3 |
|  |  | TEAD1 |
|  |  | KIAA1553 |
|  |  | CDC25B |
|  |  | RNF141 |
|  |  | ARHGEF7 |
|  |  | BMPR1A |
|  |  | RELT |
|  |  | PAIP2 |
|  |  | SP6 |
|  |  | SLC8A1 |
|  |  | C14orf126 |
|  |  | PTGDR |
|  |  | CIAO1 |
|  |  | ATP8B2 |
|  |  | FAM98A |
|  |  | BTG1 |
|  |  | YTHDF3 |
|  |  | GEMIN8 |
|  |  | NAT8L |
|  |  | DNAJC5 |
|  |  | GATA6 |
|  |  | CABP1 |
|  |  | RFXAP |
|  |  | FAS |
|  |  | AFG3L2 |
|  |  | TPM3 |
|  |  | GLRA2 |
|  |  | LCOR |
|  |  | DMRT3 |
|  |  | CYSLTR2 |
|  |  | SLC25A16 |
|  |  | DDAH1 |
|  |  | HYOU1 |
|  |  | OTUD4 |
|  |  | GCC2 |
|  |  | YWHAQ |
|  |  | C20orf177 |
|  |  | ARL13B |
|  |  | ABHD2 |
|  |  | PLCL2 |
|  |  | FAM76B |
|  |  | NUP153 |
|  |  | MUC7 |
|  |  | CLK2 |
|  |  | TTYH3 |
|  |  | C2orf44 |
|  |  | THRB |
|  |  | ZNF187 |
|  |  | AGPAT3 |
|  |  | CNOT1 |
|  |  | RAB14 |
|  |  | GALNT5 |
|  |  | CAMK2D |
|  |  | EXOC2 |
|  |  | SLC25A44 |
|  |  | HOXB3 |
|  |  | DPYD |
|  |  | LIF |
|  |  | CCM2 |
|  |  | SDC2 |
|  |  | RASSF5 |
|  |  | C6orf166 |
|  |  | ARID5B |
|  |  | LRRTM3 |
|  |  | ARL4C |
|  |  | SCD5 |
|  |  | FGF1 |
|  |  | CHRM1 |
|  |  | PLD1 |
|  |  | GCA |
|  |  | EPHA4 |
|  |  | PRKCD |
|  |  | AGGF1 |
|  |  | C11orf58 |
|  |  | ASB3 |
|  |  | SUV420H1 |
|  |  | ENAH |
|  |  | SNX10 |
|  |  | NBPF3 |
|  |  | NKTR |
|  |  | ZNF366 |
|  |  | ASAH1 |
|  |  | ZNF350 |
|  |  | MTR |
|  |  | FN1 |
|  |  | RSU1 |
|  |  | ABCC4 |
|  |  | ARIH2 |
|  |  | SNN |
|  |  | ZNF80 |
|  |  | C9orf6 |
|  |  | SSRP1 |
|  |  | HOXB5 |
|  |  | CBLB |
|  |  | EMID1 |
|  |  | MLL3 |
|  |  | ZFP36 |
|  |  | RAD1 |
|  |  | TTC3 |
|  |  | EEPD1 |
|  |  | FYN |
|  |  | SH3GL3 |
|  |  | VEGFC |
|  |  | RPN1 |
|  |  | LRBA |
|  |  | SERP1 |
|  |  | SOLH |
|  |  | JHDM1D |
|  |  | KIAA1147 |
|  |  | PDPK1 |
|  |  | CD2AP |
|  |  | ST3GAL6 |
|  |  | C15orf29 |
|  |  | XIAP |
|  |  | USP25 |
|  |  | PRKX |
|  |  | EIF5A2 |
|  |  | NFKBID |
|  |  | INSM2 |
|  |  | SIPA1L3 |
|  |  | ATXN3 |
|  |  | HOXA5 |
|  |  | ATP6V1A |
|  |  | ANKRD17 |
|  |  | DGKB |
|  |  | CDC42 |
|  |  | LHX8 |
|  |  | ACCN2 |
|  |  | COL19A1 |
|  |  | SETD5 |
|  |  | C1GALT1 |
|  |  | TOR2A |
|  |  | TOX |
|  |  | KLF12 |
|  |  | MAPK7 |
|  |  | GCH1 |
|  |  | DNA2 |
|  |  | HERC3 |
|  |  | ANKRD40 |
|  |  | VDAC3 |
|  |  | CSNK1D |
|  |  | TSPYL5 |
|  |  | YPEL1 |
|  |  | ITGB8 |
|  |  | STX5 |
|  |  | SOCS4 |
|  |  | ARPP-19 |
|  |  | PITPNM2 |
|  |  | SFRS8 |
|  |  | ITGA2 |
|  |  | SLC5A3 |
|  |  | RBPMS2 |
|  |  | CHST2 |
|  |  | BCOR |
|  |  | H2AFZ |
|  |  | CRISP2 |
|  |  | JAZF1 |
|  |  | ZBTB39 |
|  |  | ARHGAP10 |
|  |  | ABCA12 |
|  |  | CCNC |
|  |  | DIS3 |
|  |  | HSDL1 |
|  |  | HUNK |
|  |  | MBD2 |
|  |  | GPD1L |
|  |  | AMOTL2 |
|  |  | SEH1L |
|  |  | PDK4 |
|  |  | FAM104A |
|  |  | FOXA3 |
|  |  | RSBN1L |
|  |  | ADAMTS6 |
|  |  | TMEM32 |
|  |  | LIMK2 |
|  |  | MTX3 |
|  |  | UBE4A |
|  |  | CACNA2D3 |
|  |  | ZNF608 |
|  |  | CA7 |
|  |  | SLC7A2 |
|  |  | SMOC2 |
|  |  | KCTD20 |
|  |  | AP1GBP1 |
|  |  | MAGEF1 |
|  |  | DNAJC6 |
|  |  | ACE |
|  |  | HMGCR |
|  |  | HORMAD2 |
|  |  | CPNE8 |
|  |  | ZNF577 |
|  |  | SLC25A25 |
|  |  | CREB3L2 |
|  |  | PDZK1IP1 |
|  |  | ZNF329 |
|  |  | FOXD2 |
|  |  | FAM73B |
|  |  | SP7 |
|  |  | UBASH3B |
|  |  | RNF207 |
|  |  | D4S234E |
|  |  | NKAIN1 |
|  |  | ANKRD57 |
|  |  | CSDC2 |
|  |  | IKZF2 |
|  |  | CBFB |
|  |  | NOL4 |
|  |  | TMEM68 |
|  |  | POLS |
|  |  | SLU7 |
|  |  | FOXJ3 |
|  |  | SHE |
|  |  | GATA3 |
|  |  | PCAF |
|  |  | PM20D1 |
|  |  | KCTD4 |
|  |  | PLCH1 |
|  |  | A2BP1 |
|  |  | ZNF493 |
|  |  | NAP1L3 |
|  |  | IL10 |
|  |  | C2orf29 |
|  |  | USP31 |
|  |  | ID4 |
|  |  | WIPF2 |
|  |  | RFT1 |
|  |  | RAD9B |
|  |  | PPP3R1 |
|  |  | EIF5 |
|  |  | GNAZ |
|  |  | NLK |
|  |  | NLN |
|  |  | FASTKD5 |
|  |  | KIAA0182 |
|  |  | PIGA |
|  |  | NETO1 |
|  |  | KLHDC3 |
|  |  | ARL4D |
|  |  | MAGI2 |
|  |  | MTMR4 |
|  |  | ADORA1 |
|  |  | NECAP1 |
|  |  | TSC22D2 |
|  |  | CALU |
|  |  | TBR1 |
|  |  | ABHD13 |
|  |  | NAP1L5 |
|  |  | APOOL |
|  |  | XPO1 |
|  |  | WDR35 |
|  |  | RALA |
|  |  | CAMK2A |
|  |  | TTL |
|  |  | FNDC3A |
|  |  | ZNF346 |
|  |  | DNAJC5B |
|  |  | NECAB1 |
|  |  | SLC27A4 |
|  |  | PPIF |
|  |  | EFNB2 |
|  |  | STRBP |
|  |  | EIF2A |
|  |  | PATZ1 |
|  |  | VIP |
|  |  | SMPD3 |
|  |  | C16orf72 |
|  |  | MAP3K12 |
|  |  | TGOLN2 |
|  |  | DNAJB9 |
|  |  | PRX |
|  |  | GIN1 |
|  |  | TSPYL1 |
|  |  | CNTNAP2 |
|  |  | TRIM44 |
|  |  | PPAP2B |
|  |  | CDK5R1 |
|  |  | PDE6B |
|  |  | MRPS16 |
|  |  | QTRTD1 |
|  |  | WNK3 |
|  |  | ABHD12B |
|  |  | ZBTB10 |
|  |  | SLFN13 |
|  |  | TCEA1 |
|  |  | KHSRP |
|  |  | KLHL31 |
|  |  | LHFP |
|  |  | SLC9A4 |
|  |  | GLRX5 |
|  |  | REPS1 |
|  |  | MAP3K4 |
|  |  | ZEB2 |
|  |  | PDHX |
|  |  | PHF15 |
|  |  | C2CD2 |
|  |  | CSNK1G1 |
|  |  | SSTR1 |
|  |  | SAP30BP |
|  |  | CLEC12A |
|  |  | ZFAND3 |
|  |  | ARID4A |
|  |  | CALM3 |
|  |  | CXorf39 |
|  |  | SEC62 |
|  |  | GABRB3 |
|  |  | MS4A7 |
|  |  | GOSR2 |
|  |  | MSTN |
|  |  | TSLP |
|  |  | CAP2 |
|  |  | TP53 |
|  |  | TSPAN6 |
|  |  | ARHGEF6 |
|  |  | NCALD |
|  |  | PHF13 |
|  |  | AMOTL1 |
|  |  | SLC35A3 |
|  |  | DYRK1A |
|  |  | GTF2I |
|  |  | DYNC2LI1 |
|  |  | RBBP5 |
|  |  | BRPF3 |
|  |  | MYCBP |
|  |  | VANGL1 |
|  |  | SEMA4F |
|  |  | ELAVL1 |
|  |  | CSRP2 |
|  |  | BNIP3L |
|  |  | CAB39L |
|  |  | STAM2 |
|  |  | CDC42BPB |
|  |  | HOXC11 |
|  |  | NPTN |
|  |  | SLC46A3 |
|  |  | TET1 |
|  |  | PLEKHJ1 |
|  |  | PPM1G |
|  |  | HSD17B12 |
|  |  | CCDC85A |
|  |  | RAP2C |
|  |  | EFNA3 |
|  |  | ARF3 |
|  |  | CFDP1 |
|  |  | PAX3 |
|  |  | TMTC4 |
|  |  | RUFY3 |
|  |  | ANK3 |
|  |  | SLFN5 |
|  |  | NSUN7 |
|  |  | VGF |
|  |  | RNF8 |
|  |  | CALD1 |
|  |  | LRRC27 |
|  |  | KITLG |
|  |  | CTSO |
|  |  | RPN2 |
|  |  | RIMS4 |
|  |  | RSPO3 |
|  |  | TXN2 |
|  |  | JPH1 |
|  |  | ZNF579 |
|  |  | GRIA4 |
|  |  | SFXN4 |
|  |  | OBFC2A |
|  |  | SLC13A3 |
|  |  | LPIN1 |
|  |  | FOXO3 |
|  |  | GALNT1 |
|  |  | FZD4 |
|  |  | STX6 |
|  |  | MFHAS1 |
|  |  | GEM |
|  |  | KCNJ16 |
|  |  | MCPH1 |
|  |  | ATOH7 |
|  |  | ADAMTSL3 |
|  |  | LGR4 |
|  |  | LPHN2 |
|  |  | SESN2 |
|  |  | SNF1LK |
|  |  | RHOBTB1 |
|  |  | ZKSCAN2 |
|  |  | ABHD6 |
|  |  | SRRM2 |
|  |  | AOX1 |
|  |  | FLJ20184 |
|  |  | C2orf30 |
|  |  | FAM13A1 |
|  |  | ARSJ |
|  |  | MAL2 |
|  |  | SEMA4C |
|  |  | TXNDC6 |
|  |  | KIAA0644 |
|  |  | STARD7 |
|  |  | FBXO10 |
|  |  | CYP1B1 |
|  |  | SMAD5 |
|  |  | SLC16A10 |
|  |  | COLQ |
|  |  | MEF2C |
|  |  | TRIM50 |
|  |  | TOP1 |
|  |  | CDYL |
| miR-128 | 143 | C17orf70 |
|  |  | MPPED2 |
|  |  | AXIN1 |
|  |  | FOXQ1 |
|  |  | NDST1 |
|  |  | IGSF3 |
|  |  | TMEM132E |
|  |  | FADS1 |
|  |  | UBE2E3 |
|  |  | ARHGAP21 |
|  |  | FUBP3 |
|  |  | DCP1A |
|  |  | MLL |
|  |  | STEAP3 |
|  |  | MGAT1 |
|  |  | ULK1 |
|  |  | HAO1 |
|  |  | SPOPL |
|  |  | NIPBL |
|  |  | ZNF24 |
|  |  | LTBP1 |
|  |  | TBC1D22B |
|  |  | ECE2 |
|  |  | CLSTN2 |
|  |  | CNOT6 |
|  |  | DTX1 |
|  |  | KLF4 |
|  |  | KCNK10 |
|  |  | CCDC6 |
|  |  | FURIN |
|  |  | RIMS3 |
|  |  | PELI3 |
|  |  | SET |
|  |  | EPB49 |
|  |  | CORO1C |
|  |  | MPP2 |
|  |  | GAD2 |
|  |  | KIAA0232 |
|  |  | DIRAS2 |
|  |  | MEGF11 |
|  |  | KCNAB1 |
|  |  | FAM155A |
|  |  | MTMR10 |
|  |  | ERC2 |
|  |  | PTPN4 |
|  |  | TAF4 |
|  |  | PFKM |
|  |  | MME |
|  |  | NTRK3 |
|  |  | SLC35F3 |
|  |  | SMG1 |
|  |  | MAP4K5 |
|  |  | UPF1 |
|  |  | ELOVL4 |
|  |  | LMTK2 |
|  |  | FAM84B |
|  |  | C11orf57 |
|  |  | RERE |
|  |  | C14orf24 |
|  |  | XPR1 |
|  |  | STK24 |
|  |  | CD34 |
|  |  | NCOA5 |
|  |  | COL3A1 |
|  |  | ARHGEF11 |
|  |  | HAS3 |
|  |  | CITED2 |
|  |  | SMCR7L |
|  |  | SS18 |
|  |  | SASH1 |
|  |  | CDC14B |
|  |  | HECTD1 |
|  |  | INSM1 |
|  |  | GCC1 |
|  |  | PHF6 |
|  |  | FOXO4 |
|  |  | GTF2A2 |
|  |  | ABBA-1 |
|  |  | VPS4B |
|  |  | DAZAP2 |
|  |  | FXR2 |
|  |  | RASGEF1B |
|  |  | MXI1 |
|  |  | ZFP36L1 |
|  |  | TNPO3 |
|  |  | DCUN1D1 |
|  |  | BAZ2B |
|  |  | DDX6 |
|  |  | ELOVL6 |
|  |  | FBLN2 |
|  |  | PPP2R2A |
|  |  | PRKD1 |
|  |  | MED13 |
|  |  | IPO7 |
|  |  | SP2 |
|  |  | ARPP-21 |
|  |  | MTDH |
|  |  | CCT3 |
|  |  | LMBR1L |
|  |  | IKZF4 |
|  |  | RSBN1 |
|  |  | NAIF1 |
|  |  | OIT3 |
|  |  | SHOC2 |
|  |  | C9orf97 |
|  |  | STK35 |
|  |  | FAM123A |
|  |  | NAB1 |
|  |  | C18orf25 |
|  |  | TMEM64 |
|  |  | UBE2E2 |
|  |  | MIPOL1 |
|  |  | SETD7 |
|  |  | LSM1 |
|  |  | CANX |
|  |  | NPTX1 |
|  |  | TMEFF1 |
|  |  | ADCY2 |
|  |  | TCF20 |
|  |  | TFEB |
|  |  | SIRT1 |
|  |  | GABBR2 |
|  |  | SMARCA2 |
|  |  | STRN4 |
|  |  | NCAN |
|  |  | ARID4B |
|  |  | LHFPL3 |
|  |  | CBX5 |
|  |  | C16orf70 |
|  |  | EFR3A |
|  |  | MYST2 |
|  |  | CRKL |
|  |  | GAD1 |
|  |  | E2F3 |
|  |  | FAM155B |
|  |  | ZC3H12B |
|  |  | C20orf39 |
|  |  | AMD1 |
|  |  | SYT4 |
|  |  | ARL8B |
|  |  | FLJ31818 |
|  |  | CENTG3 |
|  |  | LETMD1 |
| miR-155 | 381 | SMNDC1 |
|  |  | PTN |
|  |  | SALL1 |
|  |  | TAF5L |
|  |  | NT5DC1 |
|  |  | LCORL |
|  |  | CHD8 |
|  |  | TOMM20 |
|  |  | NUFIP2 |
|  |  | ZDHHC2 |
|  |  | EPB41L4B |
|  |  | ARVCF |
|  |  | MEX3B |
|  |  | MATR3 |
|  |  | ZNF85 |
|  |  | C19orf39 |
|  |  | CHD9 |
|  |  | SYNE2 |
|  |  | PAPOLA |
|  |  | SSH2 |
|  |  | IRF2BP2 |
|  |  | DNAJB1 |
|  |  | C18orf8 |
|  |  | C10orf12 |
|  |  | C15orf41 |
|  |  | TRPM8 |
|  |  | ACTR8 |
|  |  | CPLX4 |
|  |  | C10orf46 |
|  |  | INTS6 |
|  |  | CCR9 |
|  |  | PDCD4 |
|  |  | CEP350 |
|  |  | FBXO22 |
|  |  | GNAS |
|  |  | LGI2 |
|  |  | TSGA14 |
|  |  | STARD5 |
|  |  | GDF6 |
|  |  | PCDHA13 |
|  |  | KIAA1715 |
|  |  | FOXE1 |
|  |  | ZBTB38 |
|  |  | PCDHA4 |
|  |  | RAPH1 |
|  |  | CNTN4 |
|  |  | USP14 |
|  |  | ETNK2 |
|  |  | ARL5B |
|  |  | HECTD2 |
|  |  | CCDC41 |
|  |  | FBXO11 |
|  |  | YWHAE |
|  |  | TMEM66 |
|  |  | POLE3 |
|  |  | BDNF |
|  |  | C9orf150 |
|  |  | LSM14A |
|  |  | UBR3 |
|  |  | TIPARP |
|  |  | TRAM1 |
|  |  | TWF1 |
|  |  | C3orf23 |
|  |  | AICDA |
|  |  | MEF2A |
|  |  | RICTOR |
|  |  | SGCB |
|  |  | IGSF11 |
|  |  | FAM135A |
|  |  | AHCYL2 |
|  |  | ZNF721 |
|  |  | HAL |
|  |  | SGIP1 |
|  |  | CUGBP2 |
|  |  | COL7A1 |
|  |  | VPS36 |
|  |  | CNIH |
|  |  | DCUN1D3 |
|  |  | ARL2BP |
|  |  | DHX40 |
|  |  | ZNF320 |
|  |  | PICALM |
|  |  | MYO10 |
|  |  | MASTL |
|  |  | PCSK6 |
|  |  | DBF4 |
|  |  | PTPN2 |
|  |  | STON1 |
|  |  | PCDHA2 |
|  |  | RSPO2 |
|  |  | BCAP29 |
|  |  | USP9X |
|  |  | VAPA |
|  |  | ZNF275 |
|  |  | UBQLN2 |
|  |  | YWHAZ |
|  |  | KRCC1 |
|  |  | SH3D19 |
|  |  | PRPF39 |
|  |  | C10orf104 |
|  |  | UPP2 |
|  |  | CHAF1A |
|  |  | ZNF236 |
|  |  | MORC3 |
|  |  | CXorf23 |
|  |  | VTI1A |
|  |  | AKAP10 |
|  |  | MEIS1 |
|  |  | PSTK |
|  |  | WWC1 |
|  |  | SLA |
|  |  | ASTN2 |
|  |  | ZNF254 |
|  |  | MOBKL3 |
|  |  | SOX10 |
|  |  | CSNK1G2 |
|  |  | ZNF518B |
|  |  | HMP19 |
|  |  | LOC137886 |
|  |  | OOEP |
|  |  | LRP1B |
|  |  | XAF1 |
|  |  | HBP1 |
|  |  | 7A5 |
|  |  | SPIN4 |
|  |  | KALRN |
|  |  | USH2A |
|  |  | SPRED1 |
|  |  | KBTBD11 |
|  |  | GALC |
|  |  | GCN5L2 |
|  |  | MIDN |
|  |  | ACVR1C |
|  |  | FLJ37543 |
|  |  | ZNF468 |
|  |  | ZNF644 |
|  |  | PSIP1 |
|  |  | SLC30A7 |
|  |  | GABARAPL1 |
|  |  | PLEKHA1 |
|  |  | KCNN3 |
|  |  | LIG4 |
|  |  | SGCZ |
|  |  | IL17RB |
|  |  | AGTR1 |
|  |  | STRN3 |
|  |  | MAP3K10 |
|  |  | C21orf66 |
|  |  | C1orf103 |
|  |  | IL1RAP |
|  |  | ITK |
|  |  | PCDHA12 |
|  |  | LCA5 |
|  |  | SELI |
|  |  | KIAA1274 |
|  |  | ZIC3 |
|  |  | NR4A3 |
|  |  | PELI1 |
|  |  | ILF3 |
|  |  | LMX1A |
|  |  | FANCF |
|  |  | CSNK1A1 |
|  |  | ARID2 |
|  |  | FAM91A1 |
|  |  | GNA13 |
|  |  | FREM2 |
|  |  | WDFY1 |
|  |  | RAPGEF4 |
|  |  | TSPAN14 |
|  |  | IKIP |
|  |  | KPNA1 |
|  |  | TRIP13 |
|  |  | ZNF260 |
|  |  | ZNF761 |
|  |  | TWSG1 |
|  |  | BRD1 |
|  |  | GABRA1 |
|  |  | GLRA3 |
|  |  | MSL3L1 |
|  |  | CD109 |
|  |  | SPAST |
|  |  | MS4A3 |
|  |  | SLC4A10 |
|  |  | GLCCI1 |
|  |  | ZNF618 |
|  |  | PKN2 |
|  |  | CARHSP1 |
|  |  | ARHGAP20 |
|  |  | CACNA1C |
|  |  | ACYP1 |
|  |  | C2orf18 |
|  |  | LECT1 |
|  |  | ZNF664 |
|  |  | VEZF1 |
|  |  | BTBD3 |
|  |  | KPNA4 |
|  |  | PCDHA10 |
|  |  | H3F3A |
|  |  | SLC11A2 |
|  |  | FEM1C |
|  |  | PHF17 |
|  |  | ZNF528 |
|  |  | TYW3 |
|  |  | F2RL2 |
|  |  | MCART6 |
|  |  | PCDHA5 |
|  |  | OGN |
|  |  | KCNJ1 |
|  |  | ARHGAP18 |
|  |  | NAMPT |
|  |  | NGEF |
|  |  | HDAC4 |
|  |  | SH3TC2 |
|  |  | PLCB1 |
|  |  | KLRA1 |
|  |  | SDCBP |
|  |  | PAK7 |
|  |  | G3BP2 |
|  |  | WDFY3 |
|  |  | THUMPD3 |
|  |  | NFIX |
|  |  | SPI1 |
|  |  | SCG2 |
|  |  | C1QL2 |
|  |  | FGF7 |
|  |  | RREB1 |
|  |  | DNAJB7 |
|  |  | DENND1B |
|  |  | RAB6A |
|  |  | S1PR1 |
|  |  | CLCN5 |
|  |  | RNF146 |
|  |  | NKX3-1 |
|  |  | CTLA4 |
|  |  | ZNF451 |
|  |  | FZD5 |
|  |  | CLINT1 |
|  |  | DCLRE1A |
|  |  | SMAD1 |
|  |  | ZNF642 |
|  |  | ANKFY1 |
|  |  | FOS |
|  |  | CCPG1 |
|  |  | BTBD1 |
|  |  | SKIV2L2 |
|  |  | LRRC49 |
|  |  | CCDC82 |
|  |  | SMARCA4 |
|  |  | RBP2 |
|  |  | TP53INP1 |
|  |  | ZNF300 |
|  |  | SKI |
|  |  | AFAR3 |
|  |  | ELOVL2 |
|  |  | WDR72 |
|  |  | KLRC3 |
|  |  | TNFAIP8 |
|  |  | SF3B1 |
|  |  | ABAT |
|  |  | RBM47 |
|  |  | KIAA1333 |
|  |  | JARID2 |
|  |  | CALN1 |
|  |  | ETS1 |
|  |  | EPM2A |
|  |  | COPS3 |
|  |  | IKBKE |
|  |  | SOCS1 |
|  |  | TRIM32 |
|  |  | LRRC59 |
|  |  | RAB34 |
|  |  | ZNF160 |
|  |  | CDC73 |
|  |  | DYNC1I1 |
|  |  | ZNF273 |
|  |  | LYSMD3 |
|  |  | LNX2 |
|  |  | PCDHA3 |
|  |  | KIAA1468 |
|  |  | SOX1 |
|  |  | ZNF507 |
|  |  | USP48 |
|  |  | MBNL3 |
|  |  | MAFB |
|  |  | SOX6 |
|  |  | INPP5D |
|  |  | FAM133A |
|  |  | PCDH9 |
|  |  | DET1 |
|  |  | ZNF626 |
|  |  | GPR85 |
|  |  | SP3 |
|  |  | C3orf18 |
|  |  | SUFU |
|  |  | UBQLN1 |
|  |  | BACH1 |
|  |  | RBMS3 |
|  |  | C7orf31 |
|  |  | TMEM194A |
|  |  | C3orf58 |
|  |  | GLIPR1 |
|  |  | SYPL1 |
|  |  | KBTBD2 |
|  |  | ZMYM2 |
|  |  | EHD1 |
|  |  | MPHOSPH9 |
|  |  | HBS1L |
|  |  | PCDHA1 |
|  |  | SNX1 |
|  |  | ZNF431 |
|  |  | HNRNPA3 |
|  |  | OLFML3 |
|  |  | S100PBP |
|  |  | PCDHA8 |
|  |  | KLHL5 |
|  |  | CD47 |
|  |  | GPRIN3 |
|  |  | STAC |
|  |  | C18orf1 |
|  |  | FBXO48 |
|  |  | CHURC1 |
|  |  | BOC |
|  |  | RAB1A |
|  |  | SSX2IP |
|  |  | GPM6B |
|  |  | IRF8 |
|  |  | ACTA1 |
|  |  | SYVN1 |
|  |  | SEMA5A |
|  |  | UST |
|  |  | ATP2B3 |
|  |  | TLE4 |
|  |  | UPF2 |
|  |  | KIAA0564 |
|  |  | TERF1 |
|  |  | F3 |
|  |  | ZNF703 |
|  |  | KCTD3 |
|  |  | MON1A |
|  |  | OLFM3 |
|  |  | RRP15 |
|  |  | BCAT1 |
|  |  | LRAT |
|  |  | WIT1 |
|  |  | CEBPB |
|  |  | SGK3 |
|  |  | FMNL2 |
|  |  | TRPS1 |
|  |  | BNC2 |
|  |  | XKR4 |
|  |  | CSF1R |
|  |  | C18orf10 |
|  |  | CA6 |
|  |  | MYO1D |
|  |  | KIAA1267 |
|  |  | RNF123 |
|  |  | MCM8 |
|  |  | NARS |
|  |  | CCDC126 |
|  |  | NDFIP1 |
|  |  | LHX9 |
|  |  | GCNT1 |
|  |  | RUFY2 |
|  |  | GJA5 |
|  |  | SERPINA10 |
|  |  | RCN2 |
|  |  | ELMOD1 |
|  |  | EIF2C4 |
|  |  | PDLIM5 |
|  |  | RPS6KA3 |
|  |  | RCBTB2 |
|  |  | TMEM178 |
|  |  | PCDHA6 |
|  |  | GOLPH3L |
|  |  | BET1 |
|  |  | CDX1 |
|  |  | KLHL24 |
|  |  | FANCD2 |
|  |  | PDE12 |
|  |  | C10orf26 |
|  |  | MYB |
